# Absolute Quantification of Apolipoproteins Following Treatment with Omega-3 Carboxylic Acids and Fenofibrate Using a High Precision Stable Isotope-labeled Recombinant Protein Fragments Based SRM Assay*[Fn FN2]

**DOI:** 10.1074/mcp.RA119.001765

**Published:** 2019-10-07

**Authors:** Andreas Hober, Fredrik Edfors, Maria Ryaboshapkina, Jonas Malmqvist, Louise Rosengren, Andrew J. Percy, Lars Lind, Björn Forsström, Mathias Uhlén, Jan Oscarsson, Tasso Miliotis

**Affiliations:** ‡ Science for Life Laboratory, KTH - Royal Institute of Technology, Stockholm, Sweden; § Department of Protein Science, KTH - Royal Institute of Technology, Stockholm, Sweden; ¶ Translational Science, Cardiovascular, Renal and Metabolism, IMED Biotech Unit, AstraZeneca, Gothenburg, Sweden; ‖ Department of Applications Development, Cambridge Isotope Laboratories, Inc., Tewksbury, MA 01876; ** Department of Medical Sciences, Uppsala University, Uppsala, Sweden; ‡‡ Global Medicines Development, Cardiovascular, Renal and Metabolism, AstraZeneca, Gothenburg, Sweden

**Keywords:** Clinical trials, assay development, targeted mass spectrometry, selected reaction monitoring, absolute quantification, apolipoproteins, fenofibrate and omega-3 carboxylic acids, NAFLD, SIS PrEST

## Abstract

Stable isotope-labeled standard (SIS) peptides are used as internal standards in targeted proteomics to provide robust protein quantification, which is required in clinical settings. However, SIS peptides are typically added post trypsin digestion and, as the digestion efficiency can vary significantly between peptides within a protein, the accuracy and precision of the assay may be compromised. These drawbacks can be remedied by a new class of internal standards introduced by the Human Protein Atlas project, which are based on SIS recombinant protein fragments called SIS PrESTs. SIS PrESTs are added initially to the sample and SIS peptides are released on trypsin digestion. The SIS PrEST technology is promising for absolute quantification of protein biomarkers but has not previously been evaluated in a clinical setting. An automated and scalable solid phase extraction workflow for desalting and enrichment of plasma digests was established enabling simultaneous preparation of up to 96 samples. Robust high-precision quantification of 13 apolipoproteins was achieved using a novel multiplex SIS PrEST-based LC-SRM/MS Tier 2 assay in non-depleted human plasma. The assay exhibited inter-day coefficients of variation between 1.5% and 14.5% (median = 3.5%) and was subsequently used to investigate the effects of omega-3 carboxylic acids (OM3-CA) and fenofibrate on these 13 apolipoproteins in human plasma samples from a randomized placebo-controlled trial, EFFECT I (NCT02354976). No significant changes were observed in the OM3-CA arm, whereas treatment with fenofibrate significantly increased apoAII and reduced apoB, apoCI, apoE and apoCIV levels. The reduction in apoCIV following fenofibrate treatment is a novel finding. The study demonstrates that SIS PrESTs can facilitate the generation of robust multiplexed biomarker Tier 2 assays for absolute quantification of proteins in clinical studies.

## Abstract (precis)

Applications of LC-SRM in clinical research are still limited. SIS PrEST are a novel class of standards added prior to trypsinization. We have developed a semi-automated sample preparation workflow and a SIS PrEST LC-SRM/MS Tier 2 assay for absolute quantification of 13 apolipoproteins in human plasma and applied it on clinical samples from the EFFECT I study. We demonstrate, for the first time, that SIS PrEST can be applied for exploratory biomarker research in clinical settings and capture drug effects.

## Graphical Abstract

**Figure:**
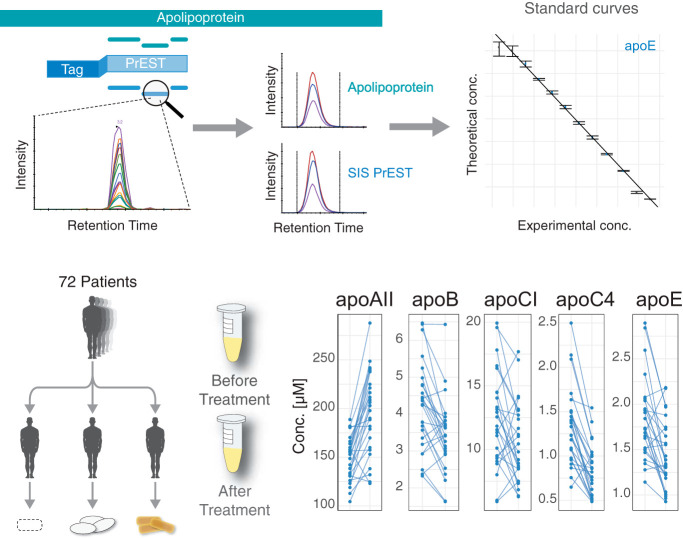

Highlights

- The first application of stable isotope-labeled recombinant protein fragments internal standards (SIS PrEST) on clinical samples.
- Development of a high-precision quantitative SIS PrEST based LC-SRM Tier 2 assay for 13 apolipoproteins.
- Semi-automated workflow enables precise and robust high-throughput sample processing.
- The assay quantifies the effects of fenofibrate and omega-3 carboxylic acids on apolipoproteins in human plasma.

The transition toward personalized medicine raises the importance of accurate and precise absolute quantification of protein biomarkers to identify patients who can benefit from targeted therapies (1, 2). Immunoassays have traditionally been the technology of choice for quantification of protein biomarkers. However, antibodies pose analytical challenges related to their specificity, selectivity and lot-to-lot variability as well as long development time and incomplete availability (3, 4). To counter these inconsistencies liquid chromatography coupled to tandem mass spectrometry (LC-MS/MS) has emerged as a powerful alternative (5). Comparatively, LC-MS/MS offers the high specificity, multiplexity, and repeatability necessary to perform biomarker assessment studies in a fit-for-purpose manner (6).

Experimentally, a protein quantification method typically uses a bottom-up workflow, which involves upfront protein denaturation and reduction/alkylation of disulfide bonds followed by tryptic digestion, solid phase extraction (SPE)^1^, and reversed-phase LC-MS/MS (7). Regarding the MS/MS, this can be accomplished in a targeted (selected or multiple reaction monitoring, SRM or MRM; parallel reaction monitoring, PRM) or data independent manner (sequential window acquisition of all theoretical mass spectra, SWATH-MS). The mechanisms of these acquisition modes are well described and have been implemented in various quantitative proteomic research studies (8).

Although quantification methods can be performed in a label-free manner, isotope-labeled standards (SISs) are recommended for absolute quantitative approaches (9, 10). These serve as internal standards (IS) and function as molecular surrogates for the endogenous proteins of interest. The most common strategy for IS deployment is to incorporate them at a specific stage of the experimental workflow. This can be accomplished at the protein or peptide level, either by incorporation of ^13^C and ^15^N isotopes at defined residues (typically l-lysine and -arginine) during protein expression or solid phase peptide synthesis, respectively. The choice of internal standard is guided by the study design as well as other factors (*e.g.* target availability). SIS proteins are added at the beginning of an analytical workflow whereas SIS peptides are added post-digestion (11). The latter approach is more convenient, but not necessarily ideal as it fails to account for the variations that arise during and preceding proteolytic digestion. This can negatively impact the accuracy and precision of protein quantification in complex biological samples such as human plasma (12). Furthermore, the conditions for the SIS and endogenous peptides will be different depending on the point of the SIS addition because of the peptide release rate during digestion (13). In contrast, the use of SIS proteins is technically superior, but practically a challenge in biomarker assessment studies because of the number of labeled proteins that would be required in preliminary screenings. Moreover, the commercial availability of SIS proteins is currently very limited. An alternative approach, which circumvents these problems, is the use of winged SIS (WiSIL, also referred to as flanked or cleavable SIS) peptides, where several residues extend the tryptic peptide on the N- and C- termini in order to better reflect the digestion conditions in the sample and improve the accuracy and precision of the MS/MS measurement (14). A further alternative is to implement trypsin digestion as a criterion for releasing the SIS peptide, as for the artificial QconCAT protein, which comprises concatenated tryptic peptides for one or more proteins and thereby combines the advantage of the WiSIL peptides with releasing multiple tryptic peptides on digestion (15, 16).

Here, we evaluate and apply an additional type of IS that is based on Stable isotope labeled Internal Standard Protein Epitope Signature Tags (SIS PrESTs) (17). SIS PrESTs are isotopically labeled recombinant protein fragments that have been produced within the Human Protein Atlas project (18) and have been used for the absolute quantification of proteins in cell lysates (19). Recently, Oeckl *et al.* presented a cross-comparison between a SIS PrEST approach and alternatives using SIS proteins, WiSIL, and SIS peptides for quantification of α-synuclein in pooled cerebrospinal fluid (20). In that study, the SIS PrEST methodology demonstrated better accuracy than WiSIL and SIS peptides compared with gold standard with known concentration of isotopically labeled alpha-synuclein, which indicated that SIS PrESTs better account for differences arising during sample preparation (including the digestion efficiency). Their data also revealed that SIS PrESTs could be accurate alternatives for SIS proteins because recombinant SIS PrESTs may mimic the structural features of proteins and therefore better account for the actual digestion conditions present in a sample. In summary, conceptual advantages and excellent technical performance make SIS PrESTs an attractive IS type for applications in clinical biomarker research. Here, we perform the first evaluation of the SIS PrEST technology for absolute quantification of protein biomarkers of samples collected in a clinical setting.

Serum lipids are transported in circulation as lipoproteins, composed of lipids and proteins, also called apolipoproteins, which are a diverse group of proteins broadly involved in turnover of lipids and innate immune response. Triglycerides (TGs) are mainly carried in very-low-density lipoproteins (VLDL) and chylomicrons, which are produced in the liver and intestine, respectively. In contrast to these TG-rich lipoprotein particles, low-density lipoproteins (LDL) and high-density lipoproteins (HDL) carry mainly cholesterol and these lipoproteins are to a large extent produced in circulation. Hypercholesterolemia, mainly elevated LDL-cholesterol (21) as well as hypertriglyceridemia are independent cardiovascular risk factors (22). Investigating a larger set of apolipoproteins may give clues to the mode of action of the drugs and potentially, in future studies, explanations to effects on hard end-points. As an example, Pechlaner *et al.* recently measured a panel of 13 apolipoproteins and demonstrated that apoCII, apoCIII and apoE are associated with cardiovascular risk (23).

Both omega-3 fatty acids and fenofibrate are indicated for the treatment of hypertriglyceridemia, but also affect cholesterol levels. Prescription grade formulations of omega-3 fatty acids given in doses of 2–4 g usually decrease serum triglycerides (TG) about 30% (24), whereas 160 or 200 mg fenofibrate decrease serum TG about 30–50%. Many studies have investigated the effects of omega-3 fatty acids and fenofibrate on circulating levels of apolipoproteins (25–43). Although apoAI, apoAII and apoB have been extensively studied, the effects of omega-3 fatty acids or fenofibrate on total circulating levels of apoCIV, apoJ (clusterin), apoAIV, apoL1, apoD or apoF have not been investigated in clinical studies to the best of our knowledge.

In this study, we developed a novel bottom-up LC-SRM/MS assay with SIS PrESTs as SIS for 13 human apolipoproteins (Table I). Preliminary development involved standard and performance optimization, along with fully automated solid phase extraction workflow for improved precision and throughput. We subsequently analyzed the 13 apolipoproteins in non-depleted plasma samples from a randomized placebo-controlled, parallel-arm study comparing the effects of 12 weeks of daily treatment with 4 g free omega-3 carboxylic acids (OM-3CA) and 200 mg fenofibrate in hypertriglyceridemic patients with non-alcoholic fatty liver disease (NAFLD) (44), a patient population with high cardiovascular risk (45).

**Table I:** Apolipoproteins quantified in this study and their SIS PrEST standards. apoB SIS PrESTs measured combined concentrations of apoB-100 and apoB-48. SIS PrEST standards marked with * were evaluated but not used as quantifiers in patient samples

Abbreviation	Protein name	Gene	UniProt ID	Ensembl ID	SIS PrEST ID
apo(a)	Apolipoprotein(a)	LPA	P08519	ENSG00000198670	HPRR2190035
apoAI	Apolipoprotein A-I	APOA1	P02647	ENSG00000118137	HPRR3450266
apoAII	Apolipoprotein A-II	APOA2	P02652	ENSG00000158874	HPRR4430020
apoAIV	Apolipoprotein A-IV	APOA4	P06727	ENSG00000110244	HPRR260124
apoB	Apolipoprotein B	APOB	P04114	ENSG00000084674	HPRR3720310*
apoB	Apolipoprotein B	APOB	P04114	ENSG00000084674	HPRR3720311
apoCI	Apolipoprotein C-I	APOC1	P02654	ENSG00000130208	HPRR3730489
apoCIV	Apolipoprotein C-IV	APOC4	P55056	ENSG00000267467	HPRR4130067
apoD	Apolipoprotein D	APOD	P05090	ENSG00000189058	HPRR2760373
apoE	Apolipoprotein E	APOE	P02649	ENSG00000130203	HPRR4340126*
apoE	Apolipoprotein E	APOE	P02649	ENSG00000130203	HPRR4200068
apoF	Apolipoprotein F	APOF	Q13790	ENSG00000175336	HPRR350023
apoJ	Clusterin	CLU	P10909	ENSG00000120885	HPRR4320626
apoLI	Apolipoprotein L1	APOL1	O14791	ENSG00000100342	HPRR350088
apoM	Apolipoprotein M	APOM	O95445	ENSG00000204444	HPRR3340379

## EXPERIMENTAL PROCEDURES

### Clinical Cohort

The design and main results of this 12-week, multicenter, randomized, placebo-controlled, double-blind, double-dummy, three-armed, parallel-group phase 2 trial, called EFFECT I, have been presented elsewhere (44). The study was conducted in accordance with the Declaration of Helsinki and the International Conference for Harmonization of Good Clinical Practice at four sites in Sweden between September 1, 2015, and May 26, 2016, and approved by the local ethics review board at Uppsala University Hospital, Uppsala, Sweden, registered at ClinicalTrials.gov (NCT02354976). In brief, the included patients were 40–75 years old and had a body mass index of 25–40 kg/m^2^, a serum triglyceride level of 1.7 mm (150 mg/dl) or higher, and a liver proton density fat fraction (PDFF) measured by magnetic resonance imaging (MRI) > 5.5% (ICD-10 version 2016 codes K76.0 and E78.1). Patients were excluded if they had diabetes mellitus, a history of other hepatic diseases, inability to undergo MRI scanning, and significant alcohol intake (over 14 standard drinks of 14 g pure alcohol per week). The treatments were 200 mg fenofibrate (Lipanthyl^®^ 200 mg capsule, Abbott), 4 g OM-3CA (Epanova^®^ 1 g capsule, AstraZeneca AB; Gothenburg, Sweden), or matching placebos as an oral daily dose. Patients were randomized 1:1:1 by a centralized randomization system using computer-generated numbers. One hundred seventy-one patients were screened, 78 patients were randomized and 72 patients completed the study (44). Fasting blood samples were collected as described previously (44), and K_2_-EDTA plasma was frozen at −80 °C at the AstraZeneca biobank for 2 to 3 years. Methods to analyze total cholesterol and TG as well as HDL-C and LDL-C concentrations have been described (44). Cholesterol in triglyceride-rich lipoproteins was calculated using the formula total cholesterol - (HDL-C+LDL-C). The power of the main study was based on the primary end-point of the percentage change in liver PDFF with OM3-CA relative to placebo (44).

### Healthy Blood Samples

Blood for method development was collected from 10 healthy non-obese Caucasian volunteers, 5 female and 5 male donors, and subsequently processed to plasma (K_2_-EDTA). Informed consent was obtained from all subjects and the study was performed in accordance with local ethical regulations following approval from the regional ethics committee “Regionala Etikprövningsnämnden i Göteborg” in Gothenburg, Sweden.

### Total Protein Quantification of Human Plasma

The total protein concentration of the plasma pool from the healthy volunteers was determined using the Pierce BCA Protein Assay Kit (Thermo Scientific, Rockford, IL) according to the instructions by the manufacturer. The absorbance was measured with a SpectraMax M2 microplate reader (Molecular Devices; Sunnyvale, CA). The total protein concentration of the pooled plasma sample was determined to 68 μg/μl, with a CV value of 5.9% based on five measurements.

### Production and Quantification of SIS PrEST Standards

The SIS PrEST standards were produced in-house and quantified within the Human Protein Atlas project as described by Edfors *et al.* (17) and shipped on dry ice to AstraZeneca (Gothenburg, Sweden). Briefly, DNA fragments were initially cloned into the expression vector pAff8c20 (46) and after that transformed into an *E. coli* strain, auxotrophic for the amino acids arginine and lysine for recombinant protein production (47). Cells containing expression vectors were cultivated in 10 ml minimal media using 100 ml shake flasks as previously described (46). Heavy isotope-labeled (^13^C and ^15^N) versions of lysine and arginine (Cambridge Isotope Laboratories; Andover, MA) were provided to the cells at 200 μg/ml to generate fully incorporated heavy protein standards. Cell cultures were harvested and the SIS PrESTs (supplemental Table S1) were purified using the N-terminal quantification tag, which include a hexahistidine tag used for purification by Immobilize Metal Affinity Chromatography (IMAC) and an Albumin Binding Protein (ABP) domain used for quantification. After purification, all isotopic SIS PrEST fragments were quantified by mass spectrometry against a non-labeled purified quantification tag standard, previously quantified by amino acid analysis. The quantification tag standard also included a C-terminal OneStrep-tag used for purification. This protein was purified using IMAC chromatography and the IMAC elution buffer was exchanged for 1xPBS (10 mm NaP, 150 mm NaCl, pH 7.3) using a PD-10 desalting column (GE Healthcare, Uppsala, Sweden). The sample was after that purified on a StrepTrap HP column (GE Healthcare) on an ÄKTA Explorer system (GE Healthcare) according to the manufacturer's protocol. All SIS PrEST fragments were quantified by mixing 1:1 with quantification tag standard, which previously had been quantified by amino acid analysis. Each sample was digested by trypsin by first reducing the proteins with 10 mm dithiothreitol (DTT) for 30 min at 56 °C. This was followed by addition of 50 mm iodoacetamide (IAA) and incubated for 20 min, shielded from light. Proteomics grade porcine trypsin (Sigma Aldrich, St Louis, MO) was added in a 1:50 enzyme to substrate ratio and the samples were incubated in a thermomixer at 37 °C. The reaction was quenched after 16 h by addition of formic acid (FA). The peptides were vacuum dried and resuspended in 3% acetonitrile (ACN), 0.1% FA, H_2_O before LC-MS/MS analysis. The concentration of each SIS PrEST was determined by averaging the ratio between three peptides (DLQAQVVESAK++, DLQAQVVESAKK++, YGVSDYHK++) originating from the ABP sequence.

### Digestion of SIS PrESTs for Assay Development

All SIS PrESTs (supplemental Table S1) were digested separately to create a peptide mixture used to identify proteotypic peptides and to optimize the corresponding SRM transitions. 10 μg of each SIS PrEST was added to 1 m ammonium bicarbonate (AMBIC, Sigma-Aldrich) and diluted to a final concentration of 50 mm AMBIC by addition of water with resistivity 18.2 mΩ·cm, produced in-house using the Milli-Q® A10 system (Merck KGaA Darmstadt; Darmstadt, Germany). Tris(2-carboxyethyl)phosphine (TCEP, Sigma-Aldrich) was added to a final concentration of 2 mm and the samples were incubated for 30 min at 56 °C. IAA (Sigma Aldrich) was added to a final concentration of 4 mm and the samples were incubated for 30 min, shielded from light. The samples were digested overnight at 37 °C by the addition of 200 ng trypsin (AB Sciex Ltd., Framingham, MA). The enzymatic reaction was stopped by addition of FA to a final concentration of 0.5%. The tryptic peptide solution was after that dried down using a sample concentrator (Christ, RVC2–25 Cdplus, Osterode, Germany). On analysis, all samples were resuspended in 100 μl of 100 mm Tris-buffer and subsequently transferred to 300 μl conical polypropylene autosampler vials (Agilent Technologies; Santa Clara, CA).

### Transition Selection and CE Optimization

To select the most appropriate SIS PrEST internal standard for each apolipoprotein target, a list of peptides and their corresponding fragment ions was generated by LC-MS/MS analysis on an Impact II^TM^ instrument (Bruker Daltonics; Hamburg, Germany) supplemented with assay parameters obtained from SRMAtlas.org (supplemental Table S2). Up to eight peptides were selected and screened for each SIS PrEST standard by injecting them individually. Approximately 1 pmol of each SIS PrEST was injected onto the LC system (Agilent 1290, Waldbronn, Germany) and separated using a Zorbax Eclipse XDB-C18 2.1 × 150 mm column packed with 1.8 μm particles with a pore size of 80 Å (Agilent Technologies), which was interfaced to a Triple Quadrupole mass spectrometer (Agilent 6490, Agilent Technologies) using the Agilent Jet Stream flow ESI source that was operated in a positive ion mode. For the initial screening, an unscheduled method with a dwell time of 50 ms for all target specific transitions was used. The collision energy (CE) optimization was done using a dynamic MRM method with delta time windows of each SRM transition set to 1 min, the gradient started at 97.3% solvent A (0.1% FA, H_2_O) and 2.7% solvent B (0.1% FA, ACN) with a constant flow rate of 0.400 ml/min. Peptides were eluted with a linear increase of solvent B from 2.7–45% over 6 min, followed by a 1 min washout as the percentage of solvent B was increased to 80%. The column was then re-equilibrated for 2 min with 2.7% of solvent B. The temperature of the column compartment was set to 50 °C and the autosampler temperature was maintained at 8 °C. Initial CE-optimization parameters were automatically generated using Skyline, software version 4.2 (48). The precursor ion of the highest intensity for each peptide together with its four most intense transitions was selected. The screened SIS PrESTs were pooled together into one equimolar pool and the CEs were further optimized using the same LC-gradient. The SRM transitions with the highest signal-to-noise ratios were saved into the final assay (supplemental Table S3) (49). An initial SIS PrEST masterbatch was made by pooling individual SIS PrESTs into an equimolar mix. This standard pool was further refined by adjusting the individual level of each SIS PrEST relative to the plasma pool so the ratio between heavy standard peptides and their corresponding endogenous counterparts would be as close to 1:1 (Heavy/Light) as possible.

### Sample Preparation

Two microliter human plasma was spiked into a pooled SIS PrEST mixture that was mixed with 12 μl 9 m Urea (Sigma-Aldrich), 300 mm Tris buffer, pH 8.0, 15 mm TCEP and incubated for 30 min at 37 °C. Subsequently, 12 μl of 75 mm IAA dissolved in water was added to the mixture and the solution was incubated for 30 min, shielded from light. The solution was subsequently diluted by adding 165 μl of 100 mm Tris (pH 8.0) in water. Trypsin (Sigma-Aldrich) was dissolved in 100 mm Tris buffer (pH 8.0) at the concentration of 0.250 μg/μl and 22 μl was added, resulting in an enzyme to substrate ratio of ∼1:15. The digestion was performed at 37 °C for 17 h and was stopped by quenching with 5 μl of concentrated FA. The solid phase extraction (SPE) was performed using the Bravo AssayMAP liquid handler robot (Agilent Technologies) using reversed-phase cartridges (RPS, #65496–60033, Agilent Technologies). The cartridges were activated with 100 μl of 0.1% trifluoroacetic acid (TFA, Sigma-Aldrich) in 50% ACN using a flow rate of 300 μl/min. The cartridges were equilibrated with 50 μl 0.1% TFA in water using a flow rate of 10 μl/min. 100 μg of tryptic peptides were loaded onto the cartridge using a flow rate of 10 μl/min. The cartridge was then washed with 50 μl of 0.1% TFA in water using a flow rate of 10 μl/min. The syringe was washed with 50 μl 0.1% TFA in 50% ACN using a flow rate of 300 μl/min and subsequently the samples were eluted in 10 μl of 0.1% TFA in 70% ACN using a flow rate of 5 μl/min. The peptides were eluted into a collection plate (Eppendorf PCR plate, Cat no. 0030129300) where the wells contained 90 μl of 0.1% FA in water, giving a ten times dilution of the eluate and a total peptide concentration of 1 μg/μl.

### Repeatability Study for the Apolipoprotein Panel

Repeatability study was conducted to assess technical assay variation under conditions as similar as possible to handling of samples from EFFECT I trial for quantification of apolipoproteins in this study. The built in “check tune” test protocol of the Agilent 6490 mass spectrometer and an injection of a plasma digest sample was conducted to evaluate instrument performance and delta retention times for each analyte prior to LC-MS/SRM analysis. The procedure described below was repeated for five consecutive days using aliquots from the same SIS PrEST masterbatch (Table II) as well as the same pooled plasma. ApoAI was not evaluated because of a shipment error. Endogenous apoAV was not detected in the human plasma samples and therefore not reported. One aliquot of pooled human plasma and masterbatch were thawed before each experiment and triplicates were made by adding 2 μl pooled human plasma to 3.8 μl of SIS PrEST masterbatch in a 1.5 ml tubes (LoBind #0030108051, Eppendorf; Hamburg, Germany). The samples were prepared as described above. After the SPE the samples were transferred from the elution plate to new tubes and stored at −80 °C until analysis. The samples were stored between one and 6 days and subsequently all subjected to LC-SRM/MS analysis on a single day and on the same instrument. A sample volume of 10 μg was injected three times for each sample and the transitions in supplemental Table S3 were monitored. The gradient started with 97.3% solvent A (0.1% FA in water) and 2.7% solvent B (0.1% FA, ACN) with a constant flow rate of 0.400 ml/min. Peptides were eluted with a linear increase of solvent B and started with an increase to 4.0% within 2 min. The content of solvent B was further increased to 14% within 13 min, to 26.1% within 7 min, to 40.5% within 3 min and up to 81% within 2 min. The content of solvent B was kept at 81% for 2 min and then gradually decreased to the initial composition within 1 min. The temperature of the column compartment was 50 °C and the autosampler temperature was maintained at 8 °C.

### Extended Repeatability Study for the Apolipoprotein Panel

The digested samples from the repeatability study were stored at −80 °C for additional 13 months, and then injected in technical triplicates on 5 consecutive days and analyzed by LC-SRM/MS using the same parameters as described above.

### Standard Curves for the Apolipoprotein Panel

A pool of SIS PrESTs (supplemental Table S4) was serially diluted into a plasma pool of five healthy volunteers in a 3-fold manner. A separate dilution was performed for the three highest points in the calibration curves by spiking of plasma into the SIL mixture in order to compensate for the limitation in volume possible to spike into human plasma. Each mixture was digested as above and analyzed using the same method used for the stability test. Standard curves were established by taking the average ratio between Heavy/Light standard peptides. Standards were established using the target bias method, and calibration points were only included if they had less than 25% CV and yielded within 25% from the expected concentration (supplemental Table S4, supplemental Fig. S1) (50).

### Comparison Between SIL Standards

Three types of proteomic standards were evaluated to assess possible digestion and quantification biases when quantifying apoAI in bottom-up proteomics settings: (1) commercial full-length SIL recombinant protein (SILU™ PROT apoAI; Sigma Aldrich), (2) SIS PrEST (detailed earlier in methods section) and (3) commercially available SIS peptide standards. The SIS peptide standards were obtained from either ThermoFisher Scientific (AQUA Ultimate grade) or New England Peptide. The three peptides employed were ATEHLSTLSE(K), THLAPYSDEL(R), and VSFLSALEEYT(K). The U-13C/15N lysine and arginine Fmoc materials, for the solid phase peptide synthesis, were from Cambridge Isotope Laboratories Inc. For both full-length apoAI and apoAI SIS PrEST (HPRR3450266), a total of 50 pmol was spiked according to the manufacturers' instructions into 1 μl plasma in quintuplicates and digested using the above described plasma digestion protocol. In addition, five samples only spiked with 1xPBS were digested together with the samples spiked with SIL protein and after digestion this set of samples was spiked with 50 pmol of each of the three SIL peptides according to the manufacturers' instructions prior to solid phase extraction (performed by the AssayMAP Bravo platform; Agilent Technologies) and LC-MRM/MS.

### SIS PrEST - Based SRM Assay In the Clinical Cohort Samples

To evaluate the effect of treatment on the apolipoprotein concentrations, the plasma samples from the clinical cohort collected during the EFFECT I study (44) were distributed into two separate 96-well plates. Pairs of patient samples from visits 1 and 4 were randomized between plates so that each treatment arm and study site had a balanced representation on each plate. Each plate included 4 additional QC samples from pooled plasma of healthy volunteers to assess potential batch to batch variability. The LC-SRM/MS was run by a person who was blinded to treatment arm. The samples were subjected to SPE and eluted off into a skirted 96-well PCR plate (Eppendorf, Cat. no. 0030129300) that was covered with a silicone sealing mat (Axygen Inc., Axymat Cat. no. AM-96-PCR-RD; Union City, CA) and subsequently transferred directly to the autosampler for analysis. We used single-point calibration, which is acceptable as long as the concentration lies within the assay linear range (51). The masterbatch was further adjusted to reach as close as possible to a 1:1 ratio between concentrations of the endogenous target proteins and their corresponding SIS PrESTs (Table II). Tryptic digestion and SPE were performed as previously described and finally 10 μg of each sample were injected into the LC-SRM/MS system using the same parameters as for the repeatability study, monitoring the transitions in supplemental Table S3. The injection order was randomized, and a blank (5% ACN, 0.1% FA) was run every tenth injection. The mass spectrometer was running a Dynamic MRM method with cycle time of 0.5 s and a maximum of 8 concurrent precursors. The mass spectrometric parameters of the Agilent 6490 triple quadrupole mass spectrometer were the following: positive ion mode, 3.5 kV capillary voltage, nozzle voltage 300 V, drying gas flow rate 15 L/min at 150 °C, nebulizer gas pressure 30 PSI at 250 °C, and Q1 and Q3 set to unit resolution.

### Experimental Design and Statistical Rationale

#### Assay Evaluation in the Repeatability Studies

Coefficients of variation were calculated based on measurements of technical triplicates aliquoted from healthy volunteer plasma pool in Skyline version 4.2. The samples were digested on 5 days. but subjected to LC-SRM/MS analysis on a single day in the repeatability and on 5 consecutive days in the extended repeatability studies. In total, 15 samples (3 aliquots by 5 occasions) and 45 injections (3 injections per sample) were analyzed for each apolipoprotein. For each apolipoprotein, median over 3 injections per sample was denoted m and intra-day CV was sd(m_sample 1 … 3_)/mean(m_sample 1 … 3_). Mean value of m for each day was denoted M = mean(m_sample 1 … 3_) on day X. Then, inter-day CV was sd(M_day 1 … 5_)/mean(M_day 1 … 5_).

#### Clinical Cohort Analysis

For each apolipoprotein, the concentration was determined in μm in each QC sample from plasma pool of healthy volunteers (4 QC samples per plate) and compared between plates using Mann-Whitney *U* test. No apolipoproteins differed significantly between the QC samples, and the absence of batch effects between plates was confirmed. Data from 72 individuals, who have completed the EFFECT I trial, were analyzed and the outcome was defined as percent change from baseline. An exploratory analysis of the effect of treatment over placebo on circulating apolipoproteins was performed with a mixed linear modeling approach because of potential correlation between response to treatment among subjects enrolled at a given study center (52, 53). Treatment was incorporated as a fixed effect and study site as a random intercept. Treatment was encoded with two variables: fenofibrate (yes *versus* no) and OM3-CA (yes *versus* no). Placebo was encoded as neither fenofibrate nor OM3-CA treatment. Interactions between treatment and study site were not considered because of the limited number of patients. Model coefficients were estimated with the iteratively reweighted generalized rank method in rlme package (54), which is robust to outliers and scenarios when model residuals deviate from the normal distribution. Spearman correlation coefficients between changes from baseline in apolipoproteins and changes from baseline in cholesterol levels in different lipoprotein particles were calculated. All tests were two-tailed. Nominal *p* values < 0.05 were considered significant. The analysis was conducted in R version 3.2.4 (55).

## RESULTS

We established a robust SIS PrEST-based SRM assay for absolute quantification of apolipoproteins. We applied this assay to assess the effects of OM3-CA and fenofibrate on apolipoprotein levels in a clinical cohort (44).

### Generation of a Multiplexed SRM Assay

**Fig. 1.**
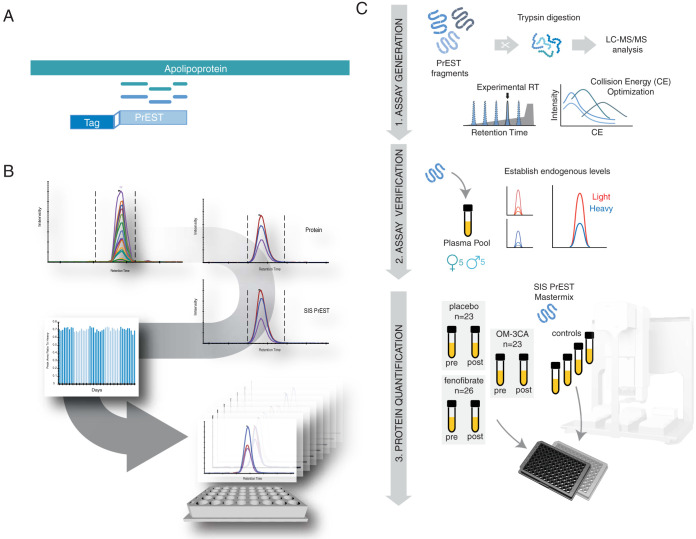
**Development of the SIS PrEST SRM-assay.** *A*, illustrates the principle of SIS PrEST as internal standards. SIS PrEST have amino acid sequence that uniquely aligns to the endogenous target protein. On tryptic digestion, proteotypic peptide fragments are released from the SIS PrEST and its target. *B*, shows the workflow for the mass spectrometric assay development including screening, transition selection and robustness studies ending with the measurement of the clinical samples. *C*, illustrates a schematic overview of the sample preparation workflow.

SIS PrESTs were initially designed to represent a unique part of each target protein (Fig. 1*A*). The enzymatic digestion by trypsin releases proteotypic peptides from the SIS PrEST and its corresponding target protein. The SIS PrEST peptides are labeled with heavy arginine and lysine to be used as internal standards, providing high precision and specificity when quantifying target proteins (19).

All SIS PrESTs were digested separately by trypsin and measured by an unscheduled SRM method monitoring the theoretical fragment ions (y- and b-ions, supplemental Table S5) representing all the theoretical proteotypic peptides originating from the SIS PrEST sequence (Fig. 1*B*). The SIS PrEST fragments for all apolipoproteins gave rise to 93 theoretical tryptic peptides, out of which 42 were successfully detected by at least three co-eluting monitored transition ions (y ions, *m*/*z* > precursor - last y ion) with uniform peak shape. All experimentally verified transitions were after that subjected to collision energy (CE) optimization performed by a scheduled SRM method and the top five most intense fragment ions were selected for each peptide respectively and further evaluated in human plasma background.

### Quantification of the Apolipoproteins in Human Plasma

The CE-optimized transitions were evaluated in a human plasma background by multiple injections using scheduled LC-SRM/MS analysis. Up to four of the most intense fragment ions without any interferences were selected based on the proposal made by Lange *et al.* (56). To determine the endogenous protein levels, we spiked in SIS PrESTs prior to trypsin digestion into the plasma pool from healthy volunteers and analyzed the samples by LC-SRM/MS using the scheduled SRM method. The spike in of SIS PrEST was subsequently adjusted in four steps to represent the endogenous protein level as close to 1:1 (SIS PrEST: endogenous protein) as possible. The final amount of spike in levels used for the SIS PrEST masterbatch is presented in Table II. The final assay for all apolipoproteins covered 34 peptides and 176 transitions measured with two-minute retention time windows with one quantifier and up to two qualifiers per peptide (supplemental Table S5). This allowed for the acquisition of at least fifteen data points across the eluting peptide peak with optimal sensitivity using the dynamic SRM setting of the triple quadrupole instrument.

**Table II:** The initial and final SIS PrEST masterbatches

Abbreviation	SIS PrEST ID	Concentration, μm	
		Initial masterbatch	Final masterbatch
apo(a)	HPRR2190035	0.44	0.35
apoAI	HPRR3450266	–	28.90
apoAII	HPRR4430020	35.82	1.23
apoAIV	HPRR260124	1.52	5.17
apoB	HPRR3720310	0.04	2.55
apoB	HPRR3720311	0.75	0.20
apoCI	HPRR3730489	6.40	2.17
apoCIV	HPRR4130067	0.14	0.02
apoD	HPRR2760373	3.16	0.22
apoE	HPRR4200068	2.69	0.01
apoE	HPRR4340126	0.25	0.56
apoF	HPRR350023	0.02	0.03
apoJ	HPRR4320626	0.02	0.60
apoLI	HPRR350088	0.27	0.12
apoM	HPRR3340379	0.69	0.73

### LLOQ and LOD

The linear dynamic range for each target was established by a serial dilution of a pool of standards spiked into human plasma (supplemental Table S4), and the resulting LOD and LLOQ can be seen in Table III for each protein. Triplicate dilution curves were made by diluting the pool of SIS PrESTs in plasma from healthy individuals. In order to determine the linear range of quantification for the different targets the following requirements were used to evaluate each data point: CV < 25% and a bias < 25%. The bias was calculated against the expected protein concentration, which was calculated through linear extrapolation. The lowest concentration fulfilling these criteria for each respective protein was assigned as the LLOQ and the LOD was calculated as the mean of the lower points excluded based on the previously stated criteria (50).

**Table III:** LOD and LLOQ for the final masterbatch. Detection limits were not established for apoD and apoJ due limited available material

SIS PrEST	Protein	Peptide sequence	Fragment	Molar concentration		
			Ion	LOD	LLOQ	Defined linear range
HPRR2190035	apo(a)	GTYSTTVTGR	y7	2.70E-09	2.52E-08	2.52E-08–7.08E-05
HPRR3450266	apoAI	VSFLSALEEYTK	y9	3.19E-08	2.76E-07	2.76E-07–1.01E-04
HPRR4430020	apoAII	SPELQAEAK	y5	2.24E-08	6.75E-07	6.75E-07–8.38E-05
HPRR260124	apoAIV	ISASAEELR	y7	1.19E-09	3.04E-09	3.04E-09–8.12E-05
HPRR3720311	apoB	SVSLPSLDPASAK	y9	3.81E-09	3.15E-09	3.15E-09–2.80E-05
HPRR3730489	apoCI	EFGNTLEDK	y5	2.71E-08	6.58E-07	6.58E-07–7.87E-05
HPRR4130067	apoCIV	AWFLESK	y5	5.88E-09	6.07E-08	6.07E-08–6.43E-05
HPRR2760373	apoD	CPNPPVQENFDVNK	y11	–	–	
HPRR4200068	apoE	LQAEAFQAR	y7	9.91E-10	2.75E-09	2.75E-09–8.63E-06
HPRR350023	apoF	FLVSLALR	y5	1.34E-09	1.05E-08	1.05E-08–1.31E-06
HPRR4320626	apoJ	IDSLLENDR	y7	–	–	
HPRR350088	apoLI	VAQELEEK	y7	8.80E-09	2.88E-08	2.88E-08–7.94E-05
HPRR3340379	apoM	AFLLTPR	y4	5.41E-10	6.90E-09	6.90E-09–2.20E-05

### Evaluation of Assay Repeatability

Three studies to evaluate distinct sources of variation using the final optimized SIS PrEST masterbatch were conducted. The clinical samples in EFFECT I were received and digested in several batches but analyzed in LC-SRM/MS in one run. We conducted a repeatability study to assess technical variation with such sample preparation scenario. The pool of plasma from healthy volunteers was individually prepared, digested in triplicate over a five-day period and analyzed by LC-SRM/MS on the same day as described in the Methods section. The coefficients of variation were below 10% for all measured apolipoproteins (Table IV).

**Table IV:** Repeatability study. Day refers to the day when tryptic digestion of the samples was performed

Protein	SIS PrEST ID	Peptide sequence	Fragment	Coefficient of variation, %		
			Ion	Intra-day	Inter-day	Overall
apo(a)	HPRR2190035	GTYSTTVTGR	y7	5.7	3.7	6.8
apoAII	HPRR4430020	SPELQAEAK	y5	3.3	1.7	3.7
apoAIV	HPRR260124	ISASAEELR	y7	3.9	1.7	4.2
apoB	HPRR3720311	SVSLPSLDPASAK	y9	4.9	4.5	6.7
apoCI	HPRR3730489	EFGNTLEDK	y5	7.5	3.7	8.3
apoCIV	HPRR4130067	AWFLESK	y5	4.1	2.8	4.9
apoD	HPRR2760373	CPNPPVQENFDVNK	y11	3.3	2.3	4.1
apoE	HPRR4200068	LQAEAFQAR	y7	4.7	3.6	6.0
apoF	HPRR350023	FLVSLALR	y5	5.3	2.5	5.8
apoJ	HPRR4320626	IDSLLENDR	y7	2.3	3.0	3.8
apoLI	HPRR350088	VAQELEEK	y7	5.0	2.9	5.8
apoM	HPRR3340379	AFLLTPR	y4	4.3	3.4	5.4
		Median	All	4.5	3.0	5.6
		Range	All	2.3–7.5	1.7–4.5	3.7–8.3

The extended repeatability study was performed to assess the day-to-day variation introduced by potential daily fluctuations in instrument performance as well as effects of prolonged storage time. Samples prepared and analyzed at different days exhibited comparable high quantitative precision as samples subjected for LC-SRM/MS analysis in one run (Table IV). apoJ had higher variation (total CV 15.3%) in the extended repeatability (Table V) than in the repeatability study. Concentrations of individual apolipoproteins were comparable in the repeatability and extended repeatability studies (supplemental Table S6).

**Table V:** Extended repeatability study. Day refers to the day when LC-SRM/MS was performed

Protein	SIS PrEST ID	Peptide sequence	Fragment	Coefficient of variation, %		
			Ion	Intra-day	Inter-day	Overall
apo(a)	HPRR2190035	GTYSTTVTGR	y7	3.9	3.6	5.3
apoAII	HPRR4430020	SPELQAEAK	y5	3.2	5.8	6.6
apoAIV	HPRR260124	ISASAEELR	y7	2.2	2.7	3.5
apoB	HPRR3720311	SVSLPSLDPASAK	y9	3.7	3.0	4.7
apoCI	HPRR3730489	EFGNTLEDK	y5	4.2	8.4	9.4
apoCIV	HPRR4130067	AWFLESK	y5	2.8	2.7	3.9
apoD	HPRR2760373	CPNPPVQENFDVNK	y11	3.3	3.9	5.1
apoE	HPRR4200068	LQAEAFQAR	y7	3.9	5.0	6.3
apoF	HPRR350023	FLVSLALR	y5	4.8	3.4	5.9
apoJ	HPRR4320626	IDSLLENDR	y7	4.9	14.5	15.3
apoLI	HPRR350088	VAQELEEK	y7	2.9	3.0	4.1
apoM	HPRR3340379	AFLLTPR	y4	2.8	1.5	3.2
		Median	All	3.5	3.5	5.2
		Range	All	2.2–4.9	1.5–14.5	3.2–15.3

**Fig. 2.**
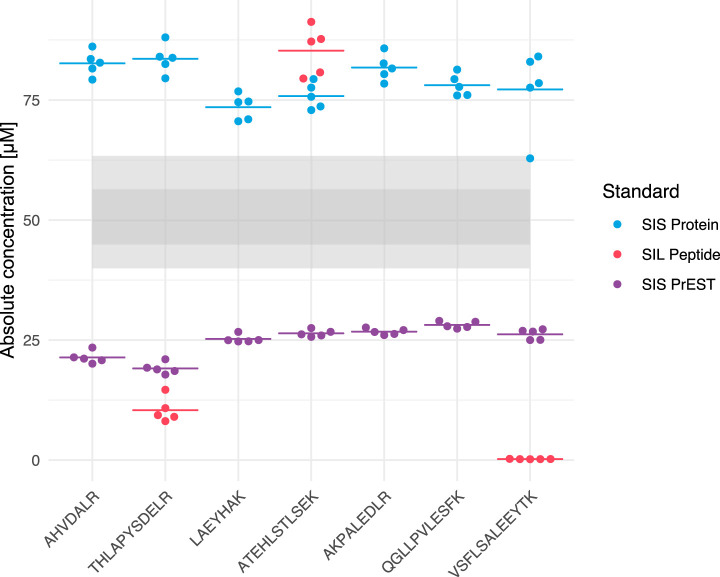
**Quantitative precision of SIS peptides, protein fragments and full-length protein.** Each color represents one standard type (SIS Protein, SIL Peptide and SIS PrEST). Each dot represents the absolute quantity in μm obtained from quantification in one replicate for the respective peptide. The median within each group is defined by one horizontal line. Each respective standard was spiked in a total amount of 50 pmol, based on the concentration provided by the suppliers, to reflect reported plasma levels. The shaded area represents the mean value ± one standard deviation within the Swedish population. The lower light gray area corresponding to the concentration in the male population and the upper light gray area corresponding the concentration in the female population, whereas the dark gray area is the overlapping concentration range for both groups.

Systematic benchmarking of all apolipoproteins against the corresponding full-length SIL protein standards was not feasible because of cost and unavailability of full-length standards for most proteins. The accuracy and precision of the SIS PrESTs was thus assessed by comparing the SIS PrEST for apoAI against a commercially available full-length SIL protein and spiked in SIS peptides that were added post digestion in order to address potential biases arising from the use of different types of internal standards. Multiple peptides exhibited excellent precision using either the SIS Protein Fragment (median CV = 3.31%) or the SIL protein standard strategy (median CV = 3.54%)(Fig. 2). Noteworthy, differences in accuracy relative literature ranges (57) can be seen between all of the investigated spiking strategies, with spiked peptides showing the largest deviation in precision (median CV = 13.1%).

Notably, none of the used standards quantified the plasma level of apoAI to the values found in literature. The targeted MS method either overestimated or underestimated the plasma level of apoAI depending on the spiked standard. However, the difference in quantity observed between different internal standards might be attributed to the difference in method used for absolute quantification of the standard itself. The SIS PrEST had been quantified against an external standard, using LC-SRM/MS, which in turn had been quantified by amino acid analysis, whereas the SIL protein was quantified by BCA, using a conversion factor established between amino acid analysis and BCA. The SIS peptides were quantified by amino acid analysis. However, both SIL protein and SIS PrEST showed great precision across multiple peptides if compared with SIS peptides, suggesting that they should be considered as preferable standards for reproducible quantification.

### Application of the SIS PrEST-based SRM Assay to Clinical Samples

**Fig. 3.**
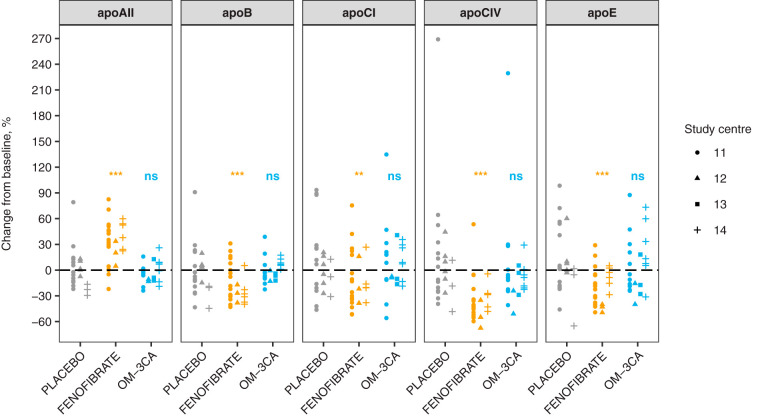
**Effects of treatment on the apolipoproteins measured with the SIS PrEST-based SRM assay.** Five apolipoproteins were significantly affected by fenofibrate, whereas none of the 13 measured apolipoproteins showed a significant response to treatment with OM-3CA. Each data point represents the within-subject change from baseline apolipoprotein concentration. Subjects enrolled at different study centers are shown with different data point shapes. The *p* values are indicated as ** for *p* < 0.01 and *** for *p* < 0.001 whereas “ns” stands for non-significant (*p* > = 0.05).

The EFFECT I clinical study has been previously described (44). In brief, the 78 randomized patients had a mean age of 60.8 years, 58% were males, BMI ranged from 25.2 to 39.2 kg/m^2^ and serum triglycerides ranged from 1.8 mmol/L to 10.1 mmol/l. The patients were randomized 1:1:1 to placebo (*n* = 26), OM-3CA (*n* = 25) and fenofibrate (*n* = 27). Out of 78 patients, 72 patients completed the study, placebo (*n* = 23), OM-3CA (*n* = 23), fenofibrate (*n* = 26). The plasma samples used for quantification of the 13 apolipoproteins using the SIS PrEST-based LC-SRM/MS assay had been frozen at −80 °C. The EFFECT I samples were all run back-to-back on the same instrument and subjected to the workflow outlined in Fig. 1*C*. The transitions corresponding to individual SIS PrEST standards exhibited high correlation with each other (supplemental Fig. S2), indicating that the apolipoprotein concentrations were not affected by the choice of transitions. All peptides showed linear dependence within the range that they were observed in the EFFECT I study (Table III). The SIS PrEST-based assay enabled precise quantification of apolipoproteins with concentrations from low nanomolar (apoF) to hundred micromolar range (apoAII) (supplemental Table S7). Fenofibrate treatment significantly increased plasma levels of apoAII (+34.4%, S.E. 3.6%, *p* value 1e-21, within-study-site correlation ICC 0.4%). Fenofibrate also significantly decreased plasma levels of apoB (-15.2%, S.E. 3.9%, *p* value 1e-4, ICC 2.7%), apoCI (-17.6%, S.E. 6.5%, *p* value 7e-3, ICC 1.3%), apoCIV (-36.4%, S.E. 4.6%, *p* value 3e-15, ICC 0.5%) and apoE (-23.9%, S.E. 6.2%, *p* value 1e-4, ICC 5.3%) (Fig. 3). Fenofibrate did not have a significant effect on apoAI, apoAIV, apoD, apoF, apoL1, apoM, apoJ or apo(a) levels. OM-3CA treatment had no significant effect on any of the analyzed apolipoproteins.

### Associations Between Changes in Apolipoproteins and Changes in Lipoprotein Fractions

We examined cross-correlations between changes from baseline in apolipoproteins and HDL-C, LDL-C and cholesterol in TG-rich particles to understand which lipoprotein fractions could represent potential sources for the observed changes. First, we investigated the associations between changes in circulating lipoprotein fractions and changes in concentrations of apolipoproteins, which were significantly affected by treatment (Table VI). Changes in apoB showed a moderate positive correlation with cholesterol in TG-rich lipoproteins in all three treatment arms, whereas apoB changes correlated more strongly with LDL-C changes in the fenofibrate and placebo groups than in the OM-3CA group (Table VI). Although apoAII changes showed a moderate positive association with changes in LDL-C and cholesterol in TG-rich lipoproteins in the placebo group, these associations were not evident in the active treatment arms. ApoE changes showed positive correlations with changes in cholesterol in TG-rich lipoproteins in mainly the OM-3CA and placebo groups. Changes in apoCIV levels showed a positive association with changes in cholesterol in TG-rich lipoproteins and LDL-C, mainly in the placebo group, but also in the fenofibrate group. Changes in apoE and apoCIV were negatively associated with changes in HDL-C in the placebo group, whereas these associations were markedly weaker in the active arms (Table VI).

**Table VI:** Lipoprotein fractions associated with the five apolipoproteins that were significantly affected by fenofibrate. The table presents Spearman correlation coefficients between percentages change from baseline. Changes in apolipoprotein concentrations were correlated with changes in lipoprotein fractions in each study arm

Correlation between	HDL-C	LDL-C	Cholesterol in TG-rich lipoproteins
**OM3-CA**			
apoAII	0.11	−0.26	0.05
apoB	−0.22	0.26	0.47
apoCI	0.05	−0.07	0.22
apoCIV	0	0.19	0.36
apoE	−0.2	−0.04	0.68
**Fenofibrate**			
apoAII	0.31	−0.23	−0.09
apoB	−0.46	0.48	0.61
apoCI	0.07	−0.15	0.14
apoCIV	−0.2	0.35	0.35
apoE	−0.02	0.08	0.36
**Placebo**			
apoAII	−0.16	0.46	0.38
apoB	−0.14	0.58	0.42
apoCI	−0.33	0.25	0.26
apoCIV	−0.49	0.47	0.6
apoE	−0.52	0.49	0.59

Next, the associations between changes in apolipoproteins, that were not significantly influenced by active treatments, were correlated with changes in lipoprotein fractions (Table VII). ApoAI changes in the active treatment arms, but not in the placebo arm, were positively correlated with changes in HDL-C. Apo(a) changes were positively correlated with LDL-C in the OM-3CA treatment group, whereas apo(a) showed a moderate negative association with cholesterol in TG-rich lipoproteins in the fenofibrate arm and HDL-C in the placebo arm. We also noted that apoL1 was negatively correlated with HDL-C in the placebo group, but not in the active treatment groups. Finally, apoD and apoM changes were positively associated with changes in LDL-C and cholesterol in TG-rich lipoproteins in the placebo group, but not in the other treatment groups. ApoF was negatively associated with cholesterol in TG-rich lipoproteins in the OM-3CA group.

**Table VII:** Lipoprotein fractions associated with the apolipoproteins that were not changed by treatment. The table presents Spearman correlation coefficients between percentages change from baseline. Changes in apolipoprotein concentrations were correlated with changes in lipoprotein fractions in each study arm

Correlation between	HDL-C	LDL-C	Cholesterol in TG-rich lipoproteins
**OM3-CA**			
apo(a)	0.15	0.37	−0.01
apoAI	0.47	0.04	−0.14
apoAIV	0.23	−0.26	0.05
apoD	0.19	−0.13	0.07
apoF	0.11	0.02	−0.40
apoJ	0.29	−0.04	−0.15
apoLI	0.25	−0.19	−0.10
apoM	0.22	−0.12	−0.07
**Fenofibrate**			
apo(a)	−0.05	0.15	−0.33
apoAI	0.40	−0.19	−0.07
apoAIV	0.12	−0.16	0.20
apoD	0.08	0.12	0.11
apoF	0.14	−0.13	0.08
apoJ	−0.14	0.30	0.17
apoLI	0.15	0.13	−0.07
apoM	0.21	−0.01	0.18
**Placebo**			
apo(a)	−0.39	−0.20	0.16
apoAI	0	0.45	0.35
apoAIV	−0.14	−0.11	0.09
apoD	−0.09	0.39	0.40
apoF	−0.19	0.07	0.07
apoJ	−0.16	0.26	0.22
apoLI	−0.53	0.18	0.28
apoM	−0.16	0.45	0.36

## DISCUSSION

The ability to measure protein biomarker panels in a clinical setting requires robust and automated assays capable of providing absolute quantitative data with high analytical rigor and sufficient throughput to scale up the analysis. Here we describe the development of an SRM assay based on SIS PrEST internal standards for the measurement of apolipoproteins in non-depleted human plasma. The flow rate of each individual syringe of the AssayMAP robot is precisely controlled in the SPE step. Furthermore, the addition of the SIS PrEST at the very beginning of the workflow and the fact that the trypsin activity released the internal standard resulted in an assay that exhibited excellent performance, which is essential for clinical application. The median inter-day CV across the measured apolipoproteins was 3.5% (extended repeatability study). The solid phase extraction step was automated using the AssayMAP robotic platform that enabled the parallel processing of up to 96 samples. Importantly, the SIS PrEST assay enabled the simultaneous quantification of multiple apolipoprotein species. Currently ∼50,000 PrEST clones (58), corresponding to more than 18,000 human proteins, are available within the Human Protein Atlas project for SIS PrEST production. Thus, it is possible to generate custom panels for absolute protein quantification for virtually any protein of interest. Also, the technology enables generation of proteins harboring specific genetic variants, which is crucial for research on personalized medicine. Overall, SIS PrEST can facilitate medical research.

In this study, we conducted a proof-of-concept application of the SIS PrEST technology in clinical settings. The primary analysis of the EFFECT I trial showed that fenofibrate reduces serum TG, LDL-C and apoCIII and increases HDL-C levels (44). The post-hoc analysis with the SIS PrEST SRM-assay showed reduced concentrations of apoB, apoCI, apoCIV and apoE and increased levels of apoAII in the fenofibrate treatment arm. However, OM-3CA treatment had no significant effect on concentrations of the analyzed apolipoproteins despite the significant reduction in serum TG that was reported in the primary analysis (44). The limitation of our study is that the EFFECT I trial was powered for the primary clinical end point (liver fat reduction) but not the post-hoc exploratory investigations. Hence, small-magnitude effects of treatment on apolipoprotein levels could have been missed. However, our results showed good agreement with previous studies (supplemental Table S8).

In various clinical intervention studies, fenofibrate treatment has shown a consistent increase in apoAII, whereas the effect on apoAI is less consistent (30–36, 59). In this study, fenofibrate treatment increased apoAII significantly, whereas no significant effect on apoAI was observed. In line with the present study, previous omega-3 fatty acid treatment studies have shown no effect on apoAI and apoAII levels (25, 26), but also a decrease in apoAI levels have been observed (29). As expected, changes in apoAI levels correlated positively with changes in HDL-C levels, whereas changes in apoAII showed weaker associations with changes in HDL-C levels but moderate positive associations with changes in apoB-containing lipoproteins.

There was no effect of any treatment on apoAIV concentrations. However, in experimental studies, the apoAIV gene expression was increased by fenofibric acid in HepG2 cells (60), whereas clofibrate reduced plasma apoAIV levels in the rat (61). These discrepancies between effects of treatment in model systems and patients highlight the necessity to conduct early proof-of-concept studies in humans. To the best of our knowledge, this is the first clinical study investigating the effects of fenofibrate or omega-3 fatty acids on apoAIV levels in patients.

In line with the previous studies (30, 32–36, 59), plasma total apoB concentrations were reduced by fenofibrate treatment; a change that mainly correlated with changes in LDL-C levels. OM3-CA treatment had no significant effect, and previous omega-3 fatty acid treatment studies have resulted in either no change (25, 26) or a decrease in apoB levels (29).

Levels of apoCI and apoCIV were reduced by fenofibrate but not by OM-3CA treatment. Fenofibrate has been shown to decrease apoCI (36), whereas to the best of our knowledge, the reduction in apoCIV is a novel finding. ApoCIV has been shown to be associated with VLDL and overexpression in mice resulted in hypertriglyceridemia (62). Interestingly, apoCIV changes showed positive associations with changes in apoB-containing lipoproteins. Therefore, one could speculate that decreased apoCIV levels may contribute to the decrease in serum TG after fenofibrate treatment.

Fenofibrate decreased apoE concentrations in line with previous studies (31, 35, 36), whereas there was no significant effect of OM3-CA in contrast to a previous study showing a decrease in apoE levels when omega-3 fatty acids were administered in combination with statins (25).

In this study, no effects on apo(a) concentrations were observed, whereas previous fenofibrate studies have shown either no change in apo(a) levels (33, 39) or an increase in apo(a) levels (42). Previous OM3-CA treatment studies either observed no change or decreased apo(a) levels (40, 41). Apo(a) is highly variable in structure and size (63). Plasminogen K4 domain type 2 may be present in 3 to 40 copies in different individuals and lead to different apo(a) forms in heterozygous individuals. The apo(a) SIS PrEST in our study specifically aligned to plasminogen K4 domain type 1, 2, 3 and 5 of apo(a) and did not map to any other human protein. We anchored the apo(a) concentration to the molar amount of the specific peptide, which is present only once in the SIS PrEST sequence. In other words, we obtained valid combined molar concentrations of apo(a) Kringle 4 Type 1, 2, 3 and 5 for each given individual, which were suitable for the purpose of the study because the outcome values (% change from baseline) were computed within-individual. However, the current SIS PrEST design of apo(a) will overestimate the apo(a) concentration because the target peptide sequence can be found in four different Kringle domains. The resulting concentration correspond to the combined amount of these Kringle domains. Hence, the apo(a) SIS PrEST design needs to be further refined for application in cross-sectional studies. Development of SIS PrEST standard that occurs only once in apo(a) sequence in every individual human and is specific to apo(a) represents a direction of future work.

Concerning the apolipoproteins that were not significantly changed by treatment in this study, including apoJ, apoM, apoLI, apoF and apoD, there is limited information about regulation in humans including treatment with fenofibrate and omega-3 fatty acids. Omega-3 fatty acid treatment has been shown to increase apoLI and apoD (38) as well as apoJ in the HDL-C fraction (39). In this study, we found a negative correlation between changes in apoLI and changes in HDL-C in the placebo group, but weaker associations in the active treatment arms. In previous studies, fibrates either did not affect apoM levels (bezafibrate) (43) or increased apoM (fenofibrate) (36).

In summary, a robust SIS PrEST-based SRM assay has been developed and shows high precision for apolipoprotein quantification. This assay was applied to a clinical cohort to investigate the differential effects of fenofibrate and OM3-CA on circulating apolipoprotein levels. To the best of our knowledge, reduction in apoCIV by fenofibrate and lack of treatment effect by either drug on apoAIV, apoJ, apoLI, apoF and apoD in hypertriglyceridemic patients have not been previously reported and therefore represent novel findings. Furthermore, we confirmed the known effects of fenofibrate that were consistent with previous studies.

## DATA AVAILABILITY

The mass-spectrometry data have been deposited at PanoramaWeb (https://panoramaweb.org/apo.url), PX ID: PXD013314.

## Supplementary Material

Supplementary Tables

Supplementary Figures

## Footnotes

^1^ The abbreviations used are:

SPE: solid phase extraction
AMBIC: ammonium bicarbonate
apo: apolipoprotein
CE: collision energy
DTT: dithiothreitol
FA: formic acid
HDL: LDL, VDL, high-, low- and very low-density lipoproteins
IAA: iodoacetamide
IS: internal standard
LC: liquid chromatography
MRI: magnetic resonance imaging
MRM: PRM, SRM, multiple, parallel and selected reaction monitoring, respectively
MS/MS: tandem mass spectrometry
NAFLD: non-alcoholic fatty liver disease
OM3-CA: omega-3 carboxylic acids
PDFF: proton density fat fraction
QconCAT: quantification concatemer
SIS: stable isotope-labeled standard
SIS PrEST: Stable isotope labeled Internal Standard Protein Epitope Signature Tag
SWATH: sequential window acquisition of all theoretical fragment ion spectra
TCEP: Tris(2-carboxyethyl)phosphine hydrochloride
TFA: trifluoroacetic acid
TG: triglyceride
WiSIL: winged Stable isotope-labeled.

## Note Added in Proof

QPrESTs have been reformulated, hence the standards used within this study are referred to as SIS PrESTs.
