# Supplementary material for: Absolute Quantification of Apolipoproteins Following Treatment with Omega-3 Carboxylic Acids and Fenofibrate Using a High Precision Stable Isotope-labeled Recombinant Protein Fragments Based SRM Assay
Source: Mol Cell Proteomics. 2019 Oct 7;18(12):2433–46. doi: 10.1074/mcp.RA119.001765 (PMC6885709; doi:10.1074/mcp.RA119.001765)
Supplement: Supplementary Figures [file 155612_0_supp_388439_pyyscz.pdf]

Supplementary Figures to

Absolute Quantification of Apolipoproteins Following Treatment with Omega-3 Carboxylic Acids and Fenofibrate Using a High Precision SIS PrEST-Based SRM Assay

Andreas Hober[1,2,#], Fredrik Edfors[1,2,#], Maria Ryaboshapkina[3], Jonas Malmqvist[3], Louise Rosengren[3], Andrew J Percy[4], Lars Lind[5], Björn Forsström[1,2], Mathias Uhlen[1,2], Jan Oscarsson[6], Tasso Miliotis[3*]

[1]Science for Life Laboratory, KTH - Royal Institute of Technology, Stockholm, Sweden

[2]Department of Protein Science, KTH - Royal Institute of Technology, Stockholm, Sweden

[3]Translational Science, Cardiovascular, Renal and Metabolism, IMED Biotech Unit, AstraZeneca, Gothenburg, Sweden

[4] Department of Applications Development, Cambridge Isotope Laboratories, Inc., Tewksbury, MA, USA

[5]Department of Medical Sciences, Uppsala University, Uppsala, Sweden

[6]Global Medicines Development, Cardiovascular, Renal and Metabolism, AstraZeneca, Gothenburg, Sweden

# These authors contributed equally to this manuscript.

* Corresponding author: Tasso.Miliotis@astrazeneca.com

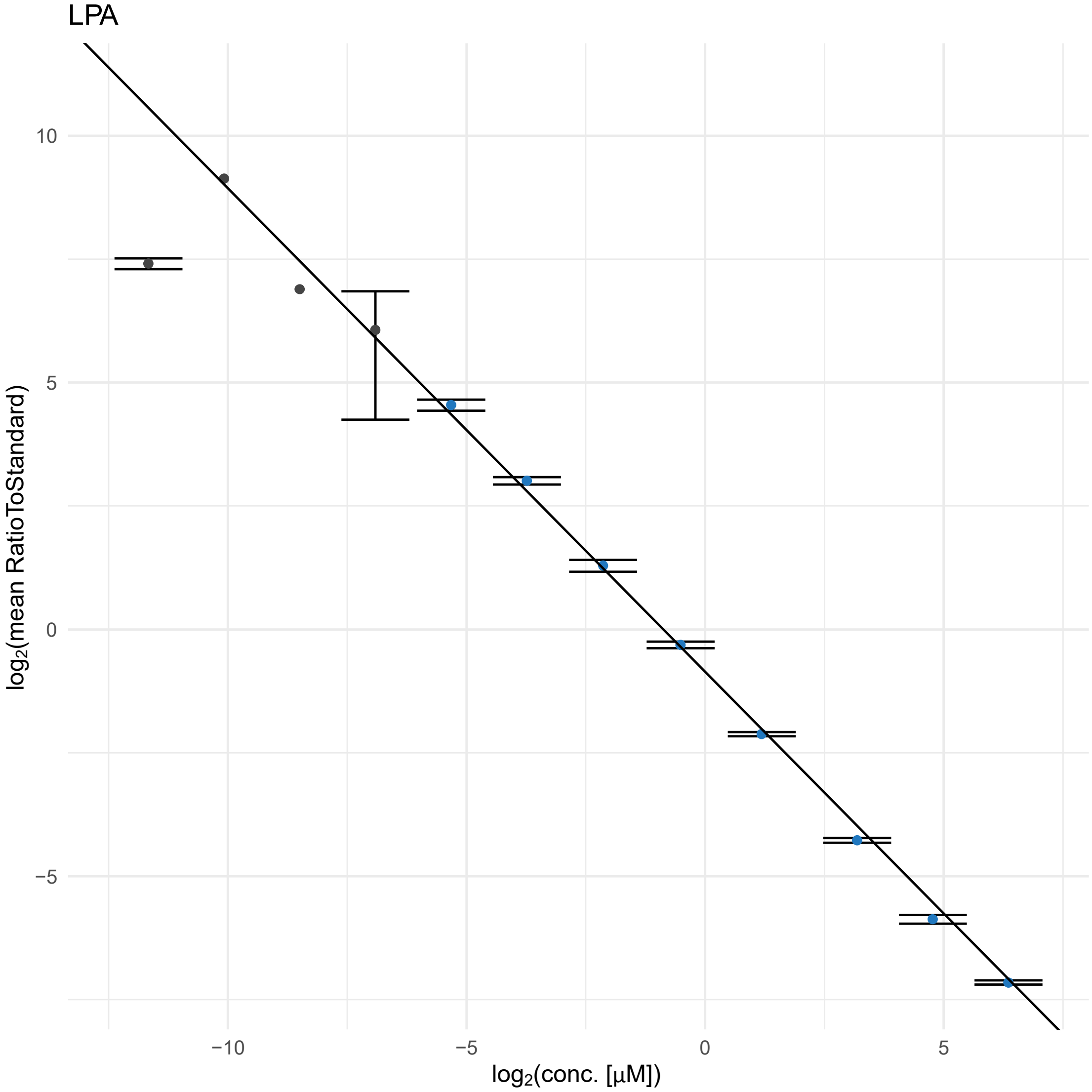

Supplementary Fig. S1a. Standard curve for apo(a) SIS PrEST HPRR2190035.

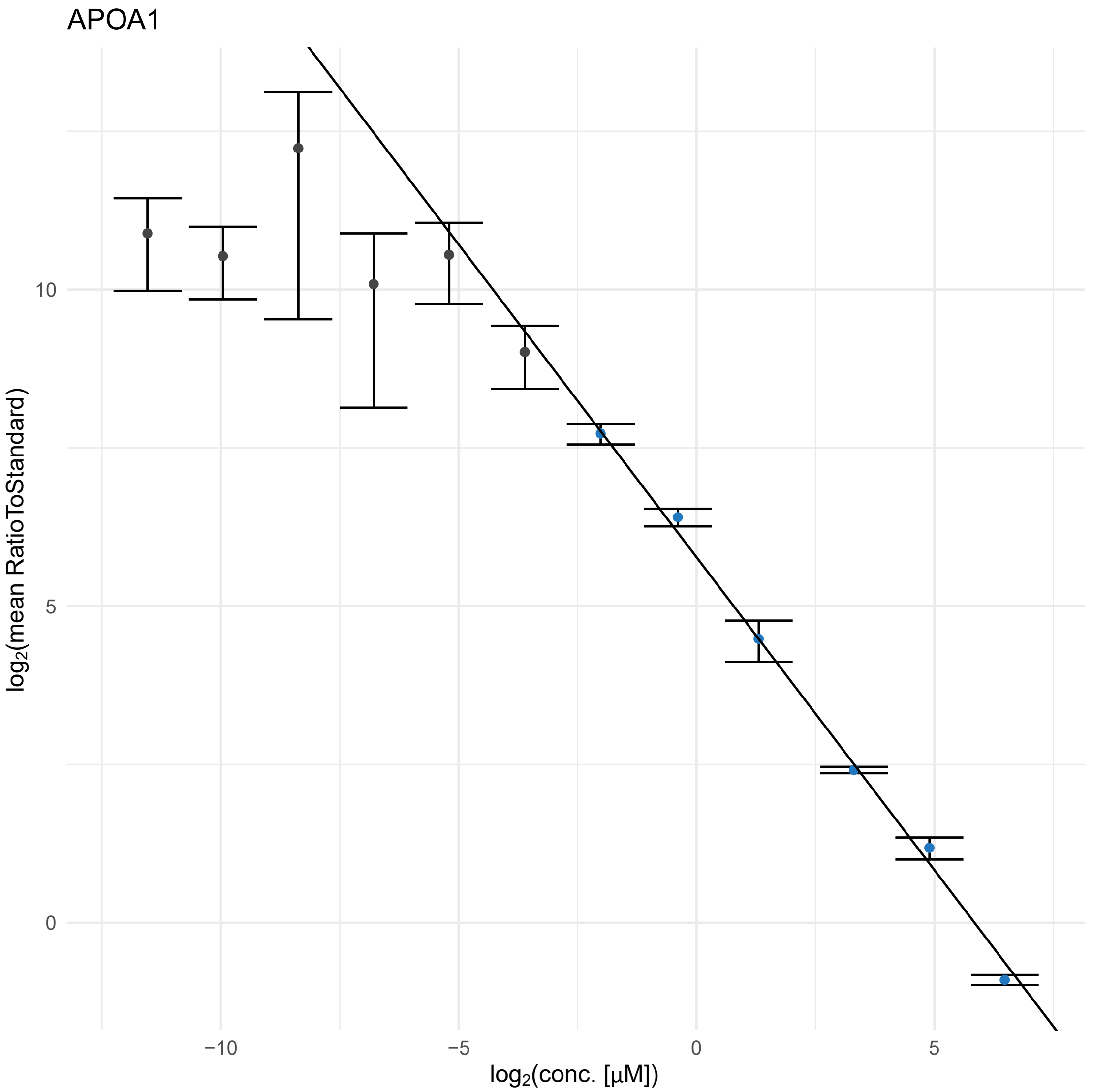

Supplementary Fig. S1b. Standard curve for apoAI SIS PrEST HPRR3450266.

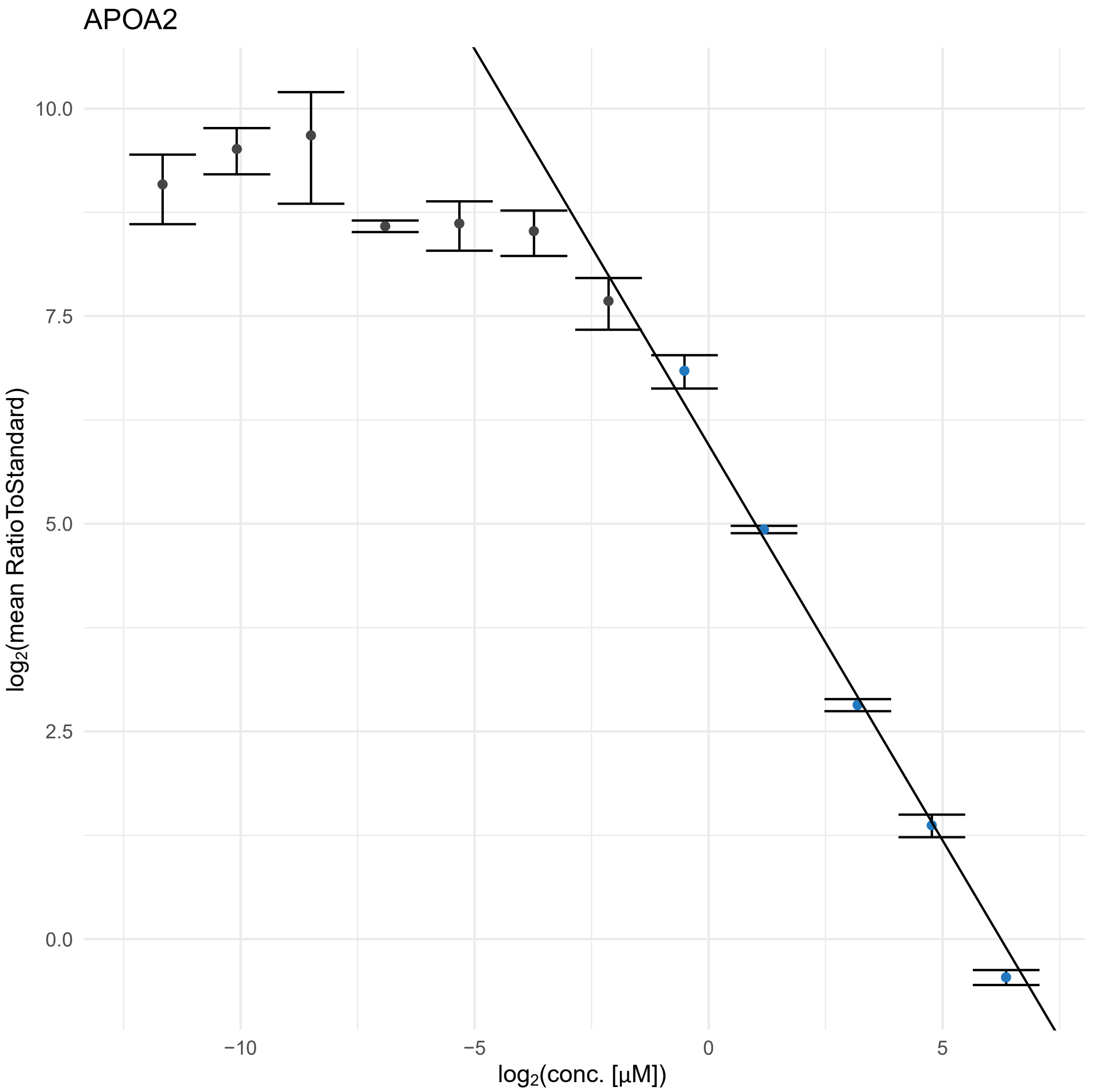

Supplementary Fig. S1c. Standard curve for apoAII SIS PrEST HPRR4430020.

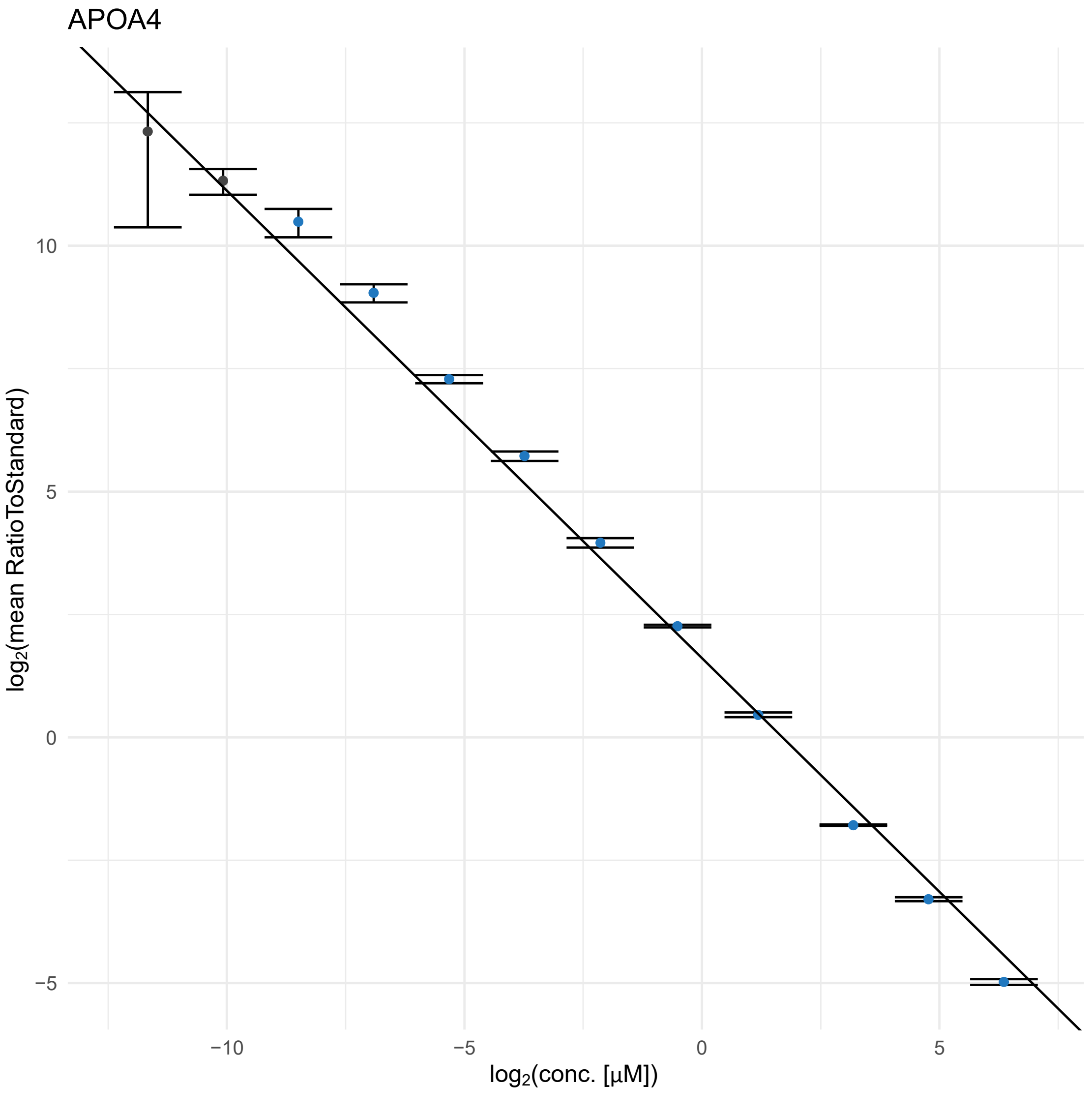

Supplementary Fig. S1d. Standard curve for apoAIV SIS PrEST HPRR260124.

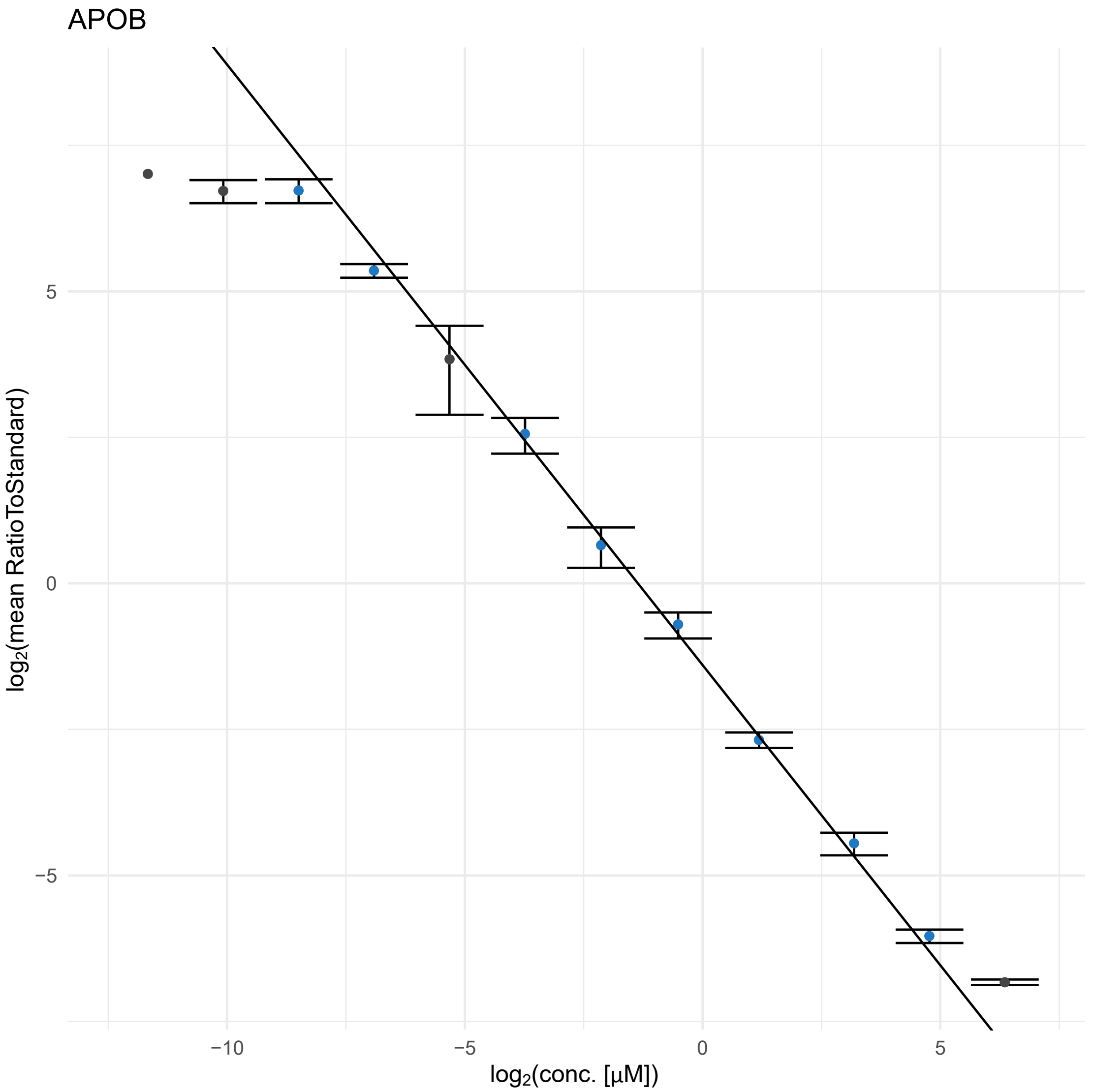

Supplementary Fig. S1e. Standard curve for apoB SIS PrEST HPRR3720311.

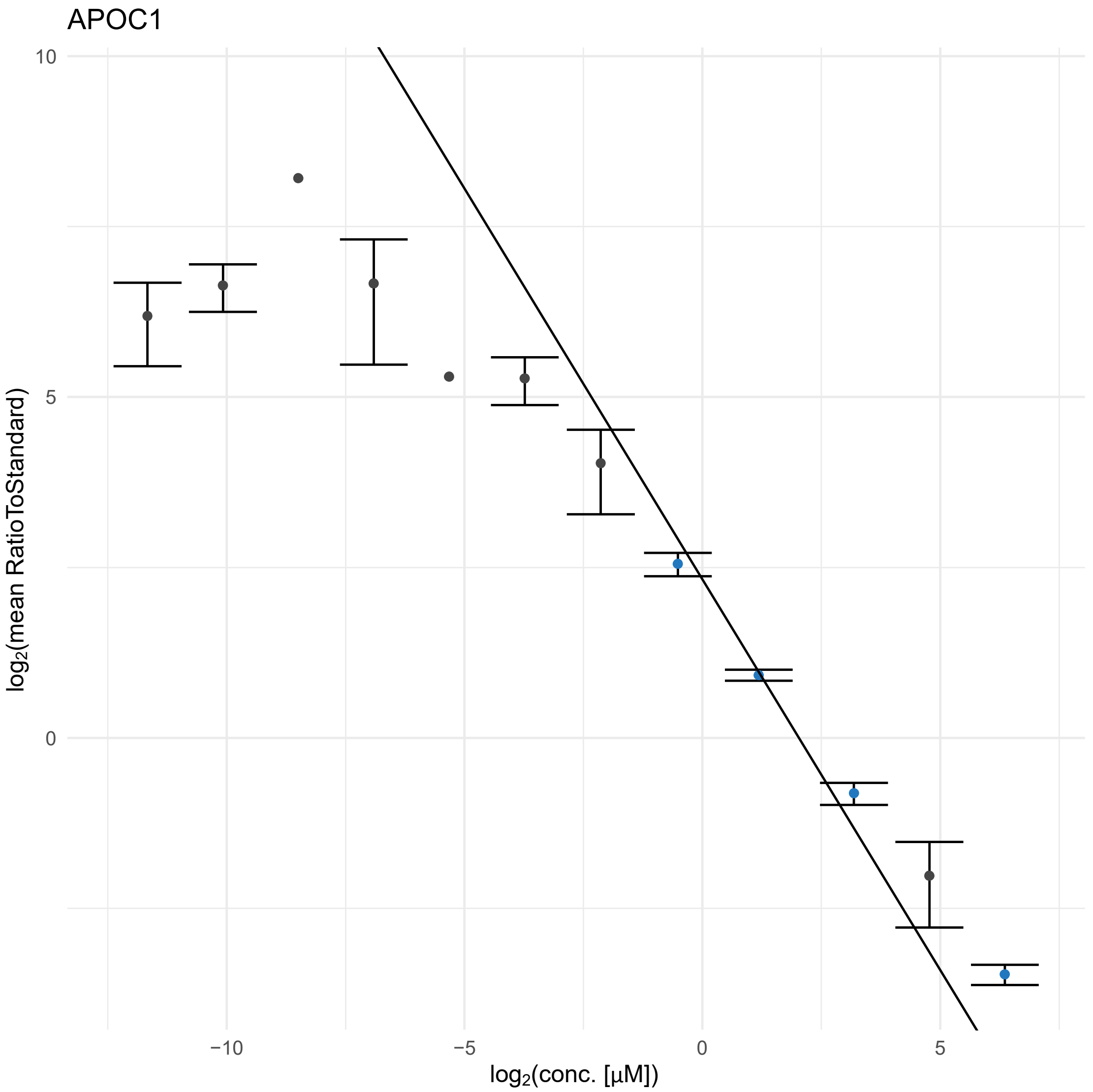

Supplementary Fig. S1f. Standard curve for apoCI SIS PrEST HPRR3730489.

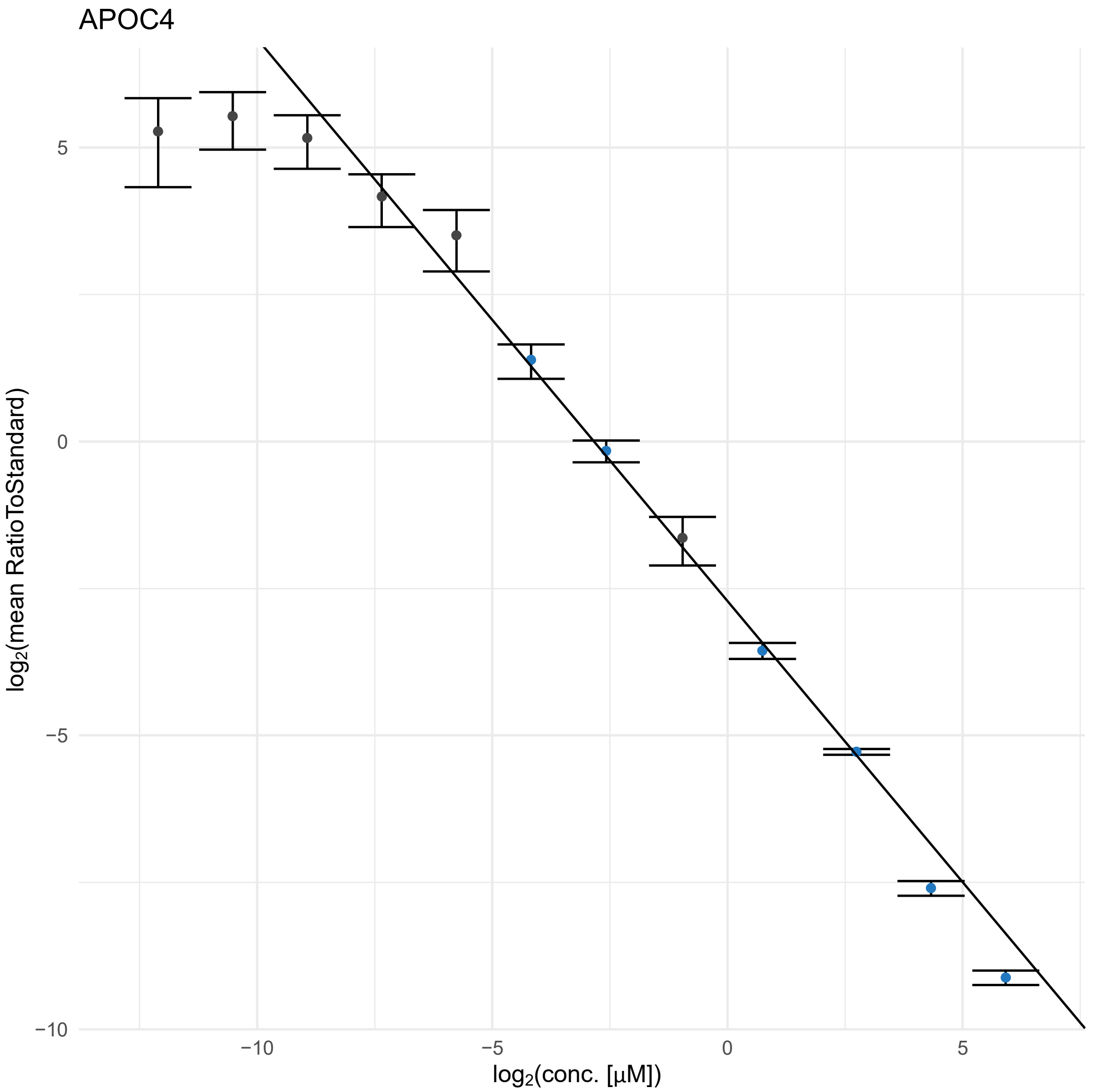

Supplementary Fig. S1g. Standard curve for apoCIV SIS PrEST HPRR4130067.

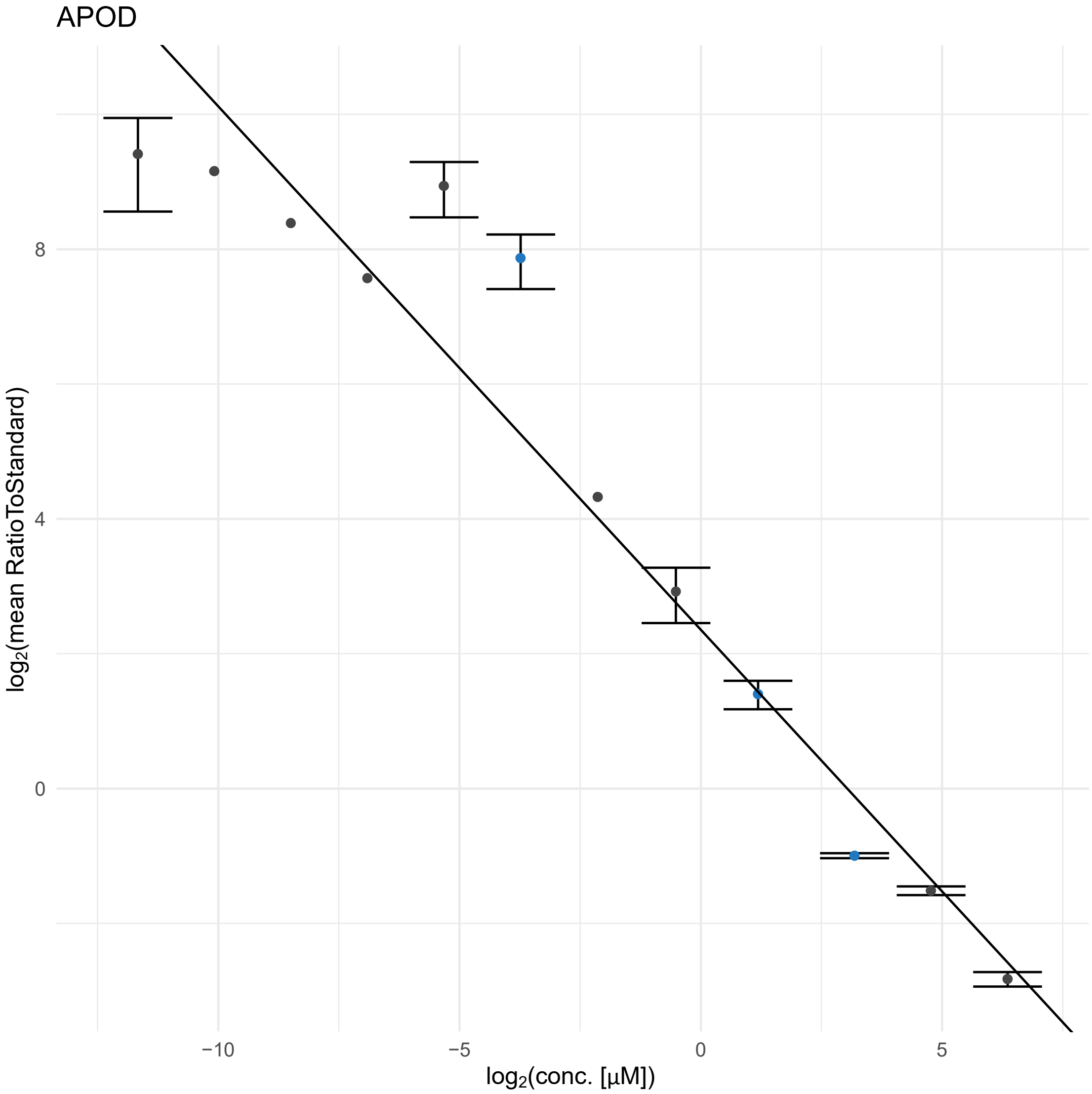

Supplementary Fig. S1h. Standard curve for apoD SIS PrEST HPRR2760373.

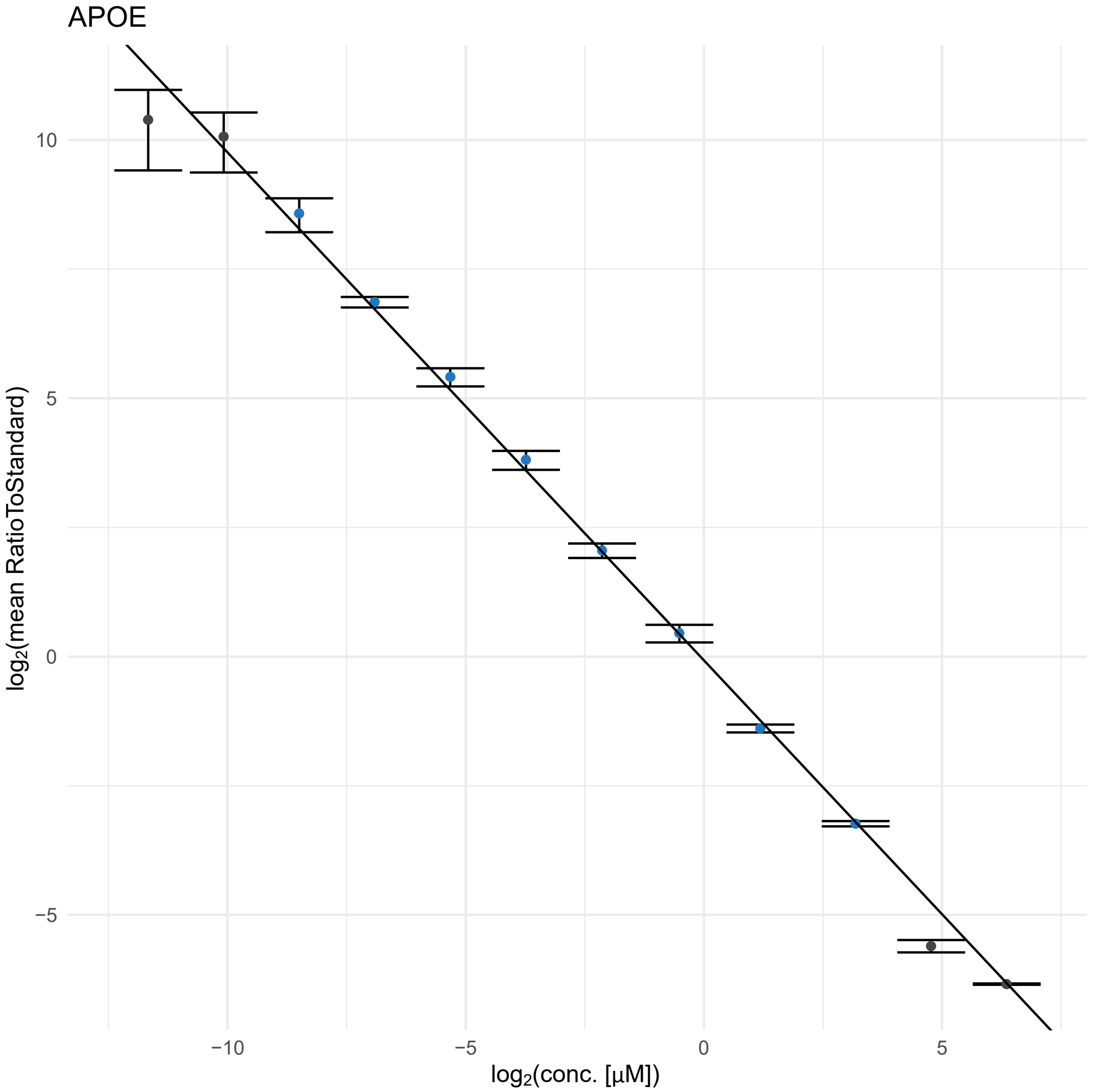

Supplementary Fig. S1i. Standard curve for apoE SIS PrEST HPRR4200068.

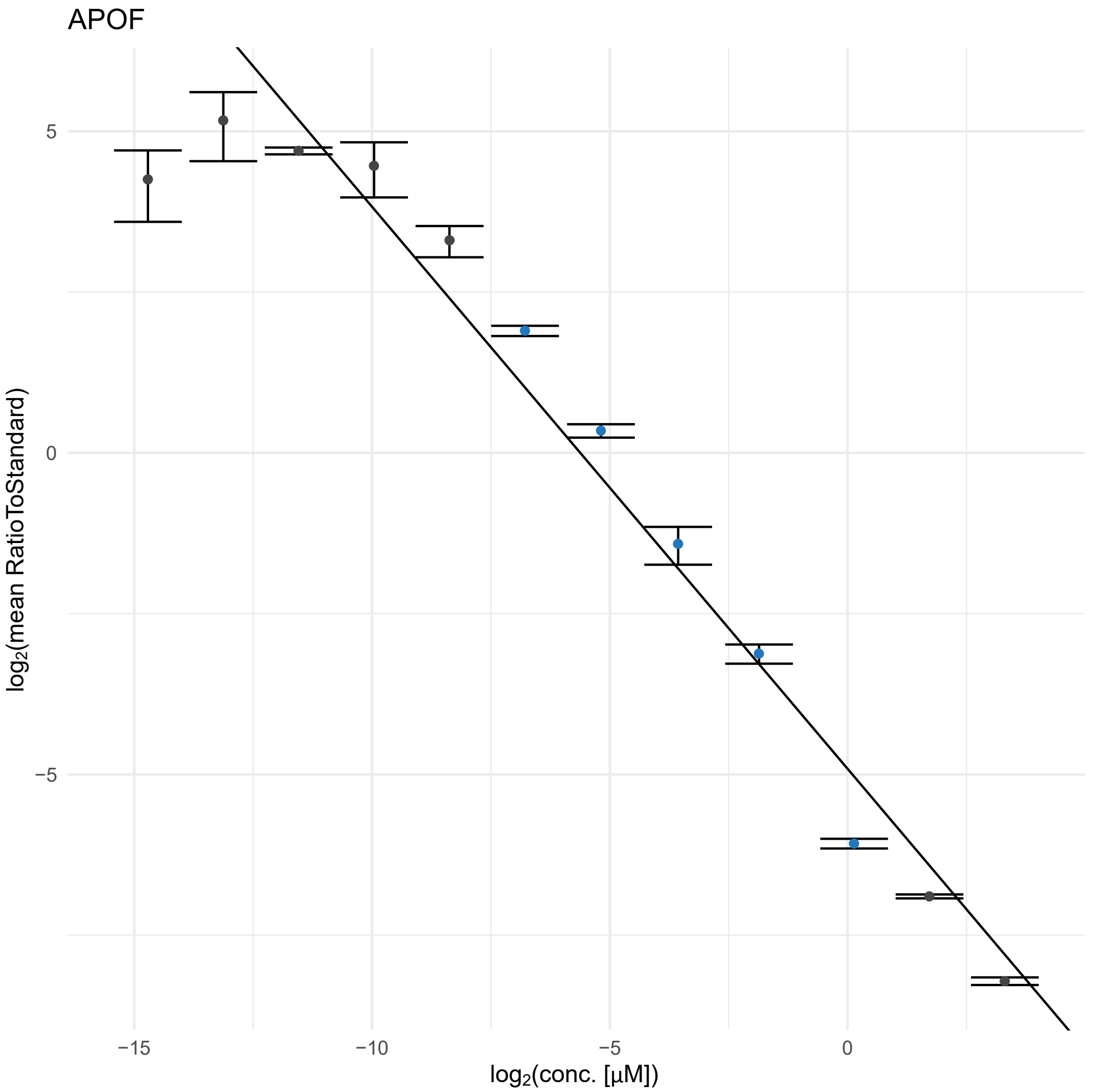

Supplementary Fig. S1j. Standard curve for apoF SIS PrEST HPRR350023.

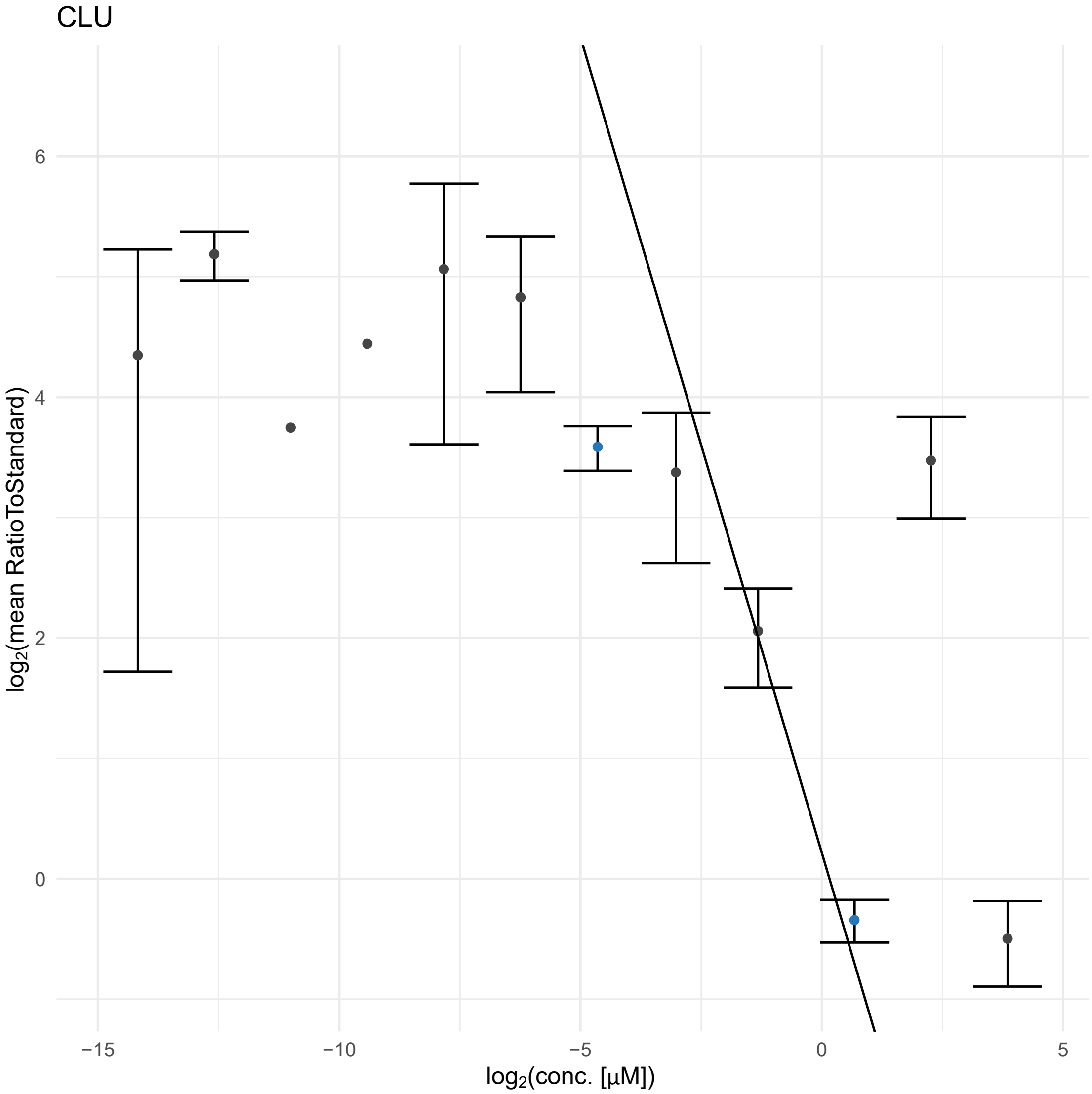

Supplementary Fig. S1k. Standard curve for apoJ SIS PrEST HPRR4320626.

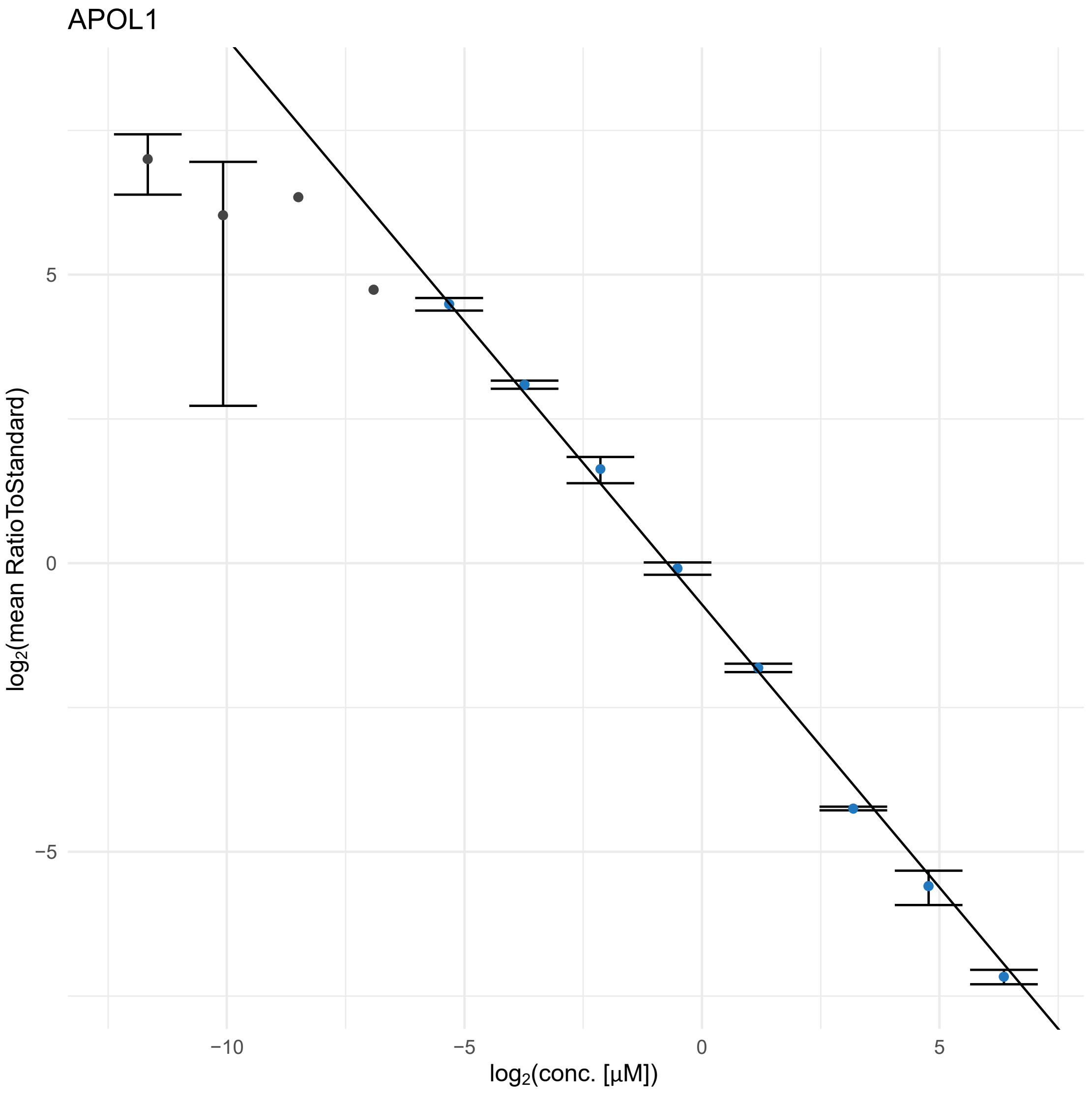

Supplementary Fig. S1l. Standard curve for apoLI SIS PrEST HPRR350088.

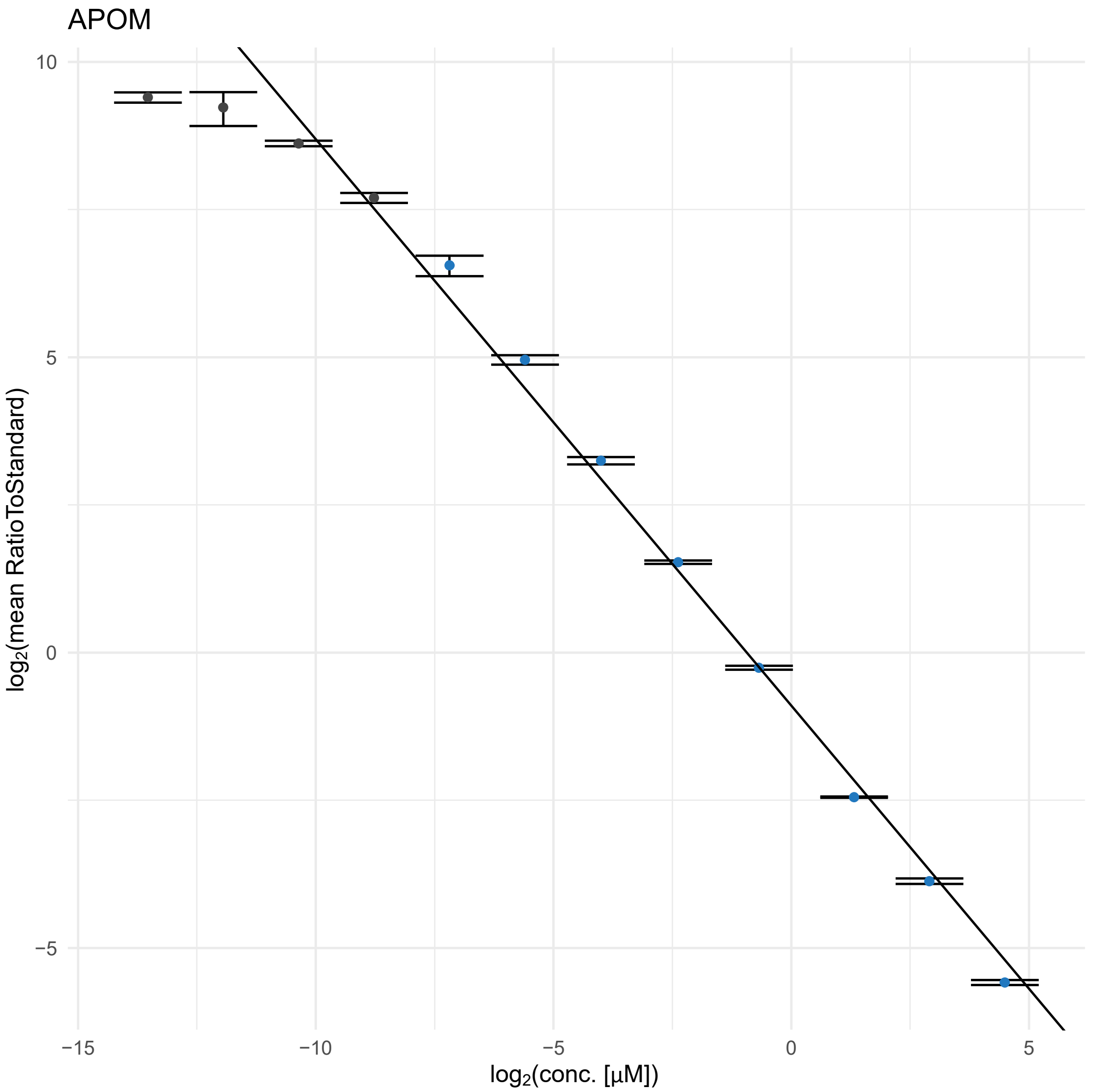

Supplementary Fig. S1m. Standard curve for apoM SIS PrEST HPRR3340379.

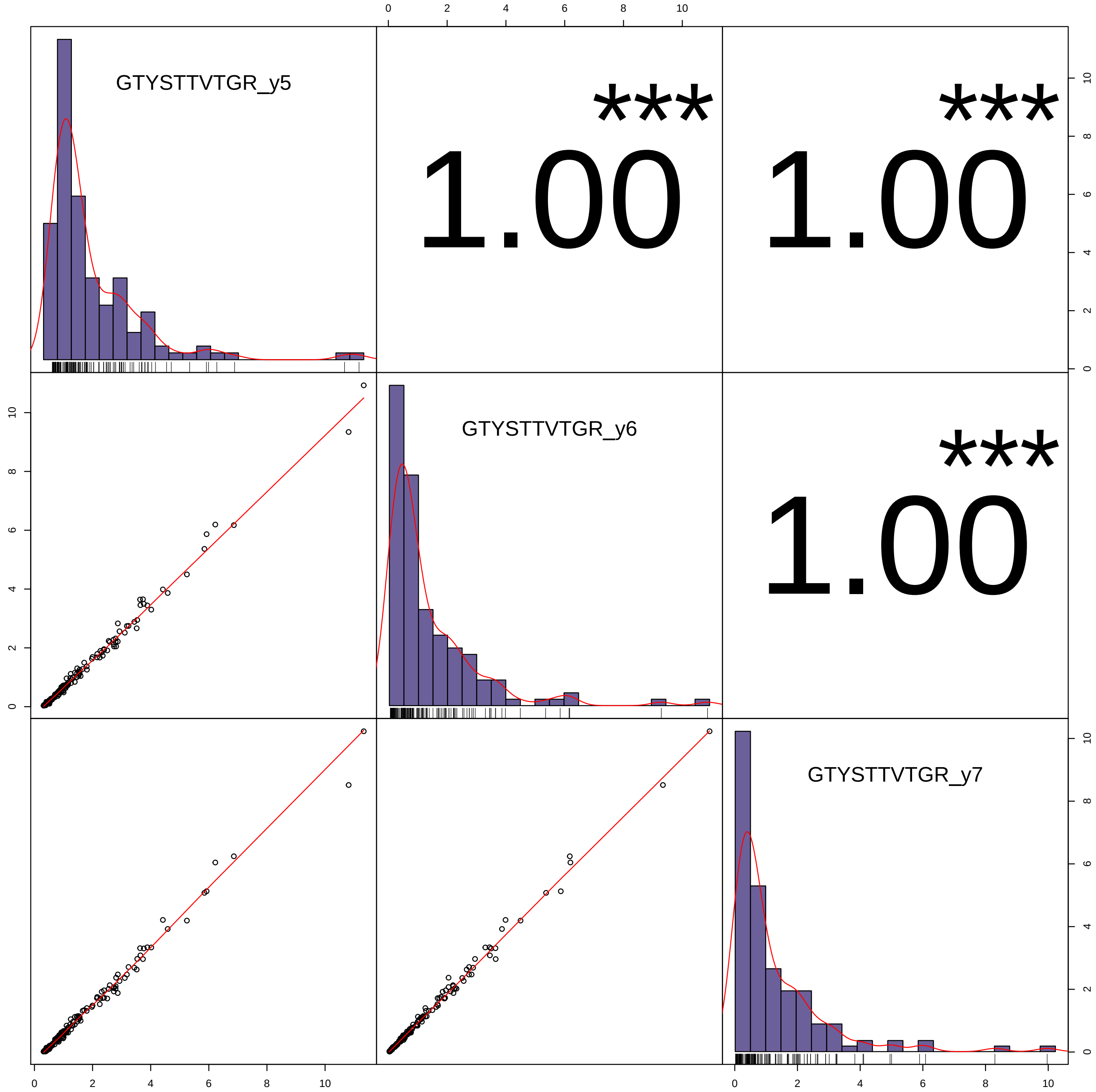

Supplementary Fig. S2a. Correlations between transitions for apo(a) QPrEST HPRR2190035.

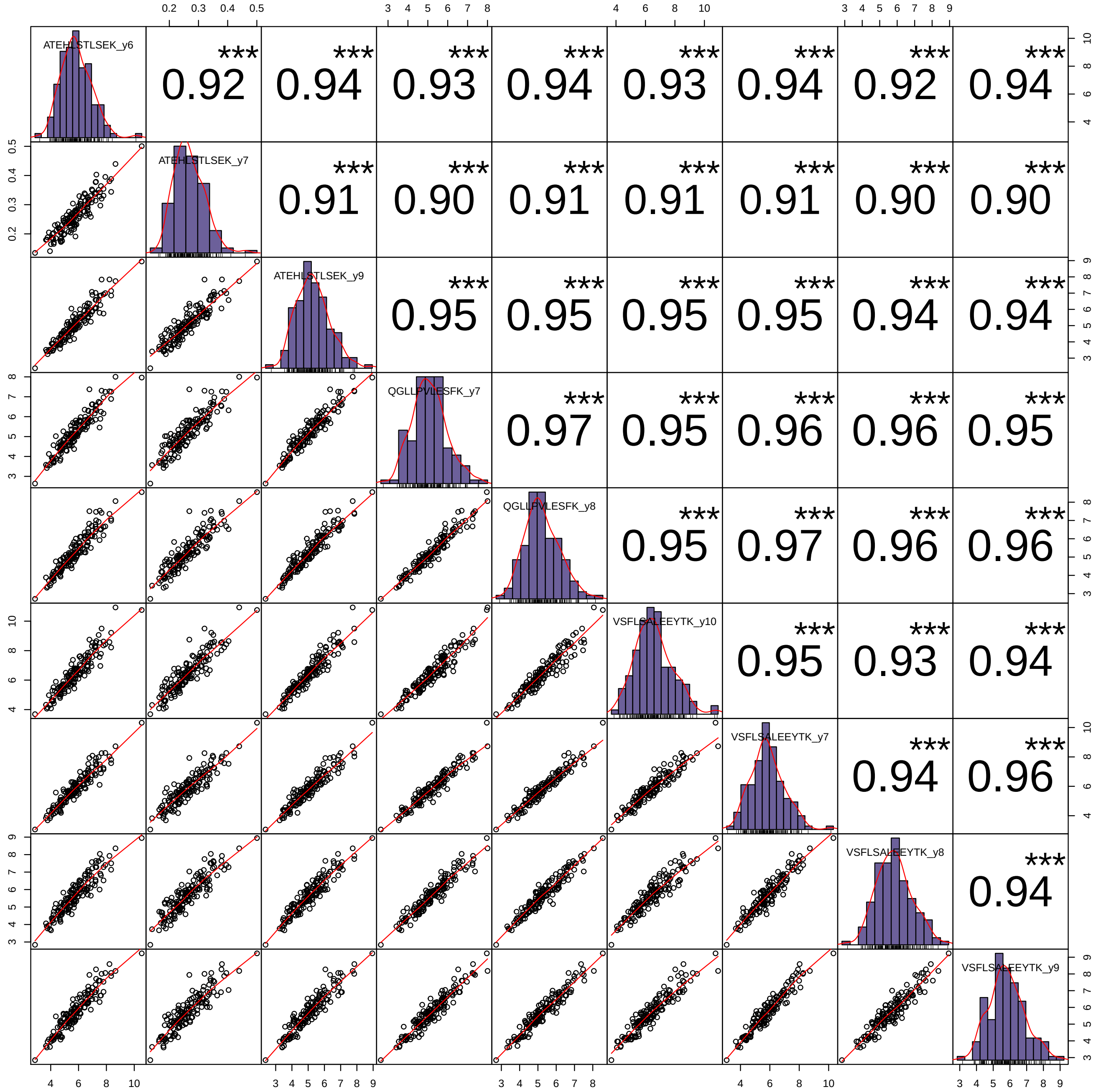

Supplementary Fig. S2b. Correlations between transitions for apoAI QPrEST HPRR3450266.

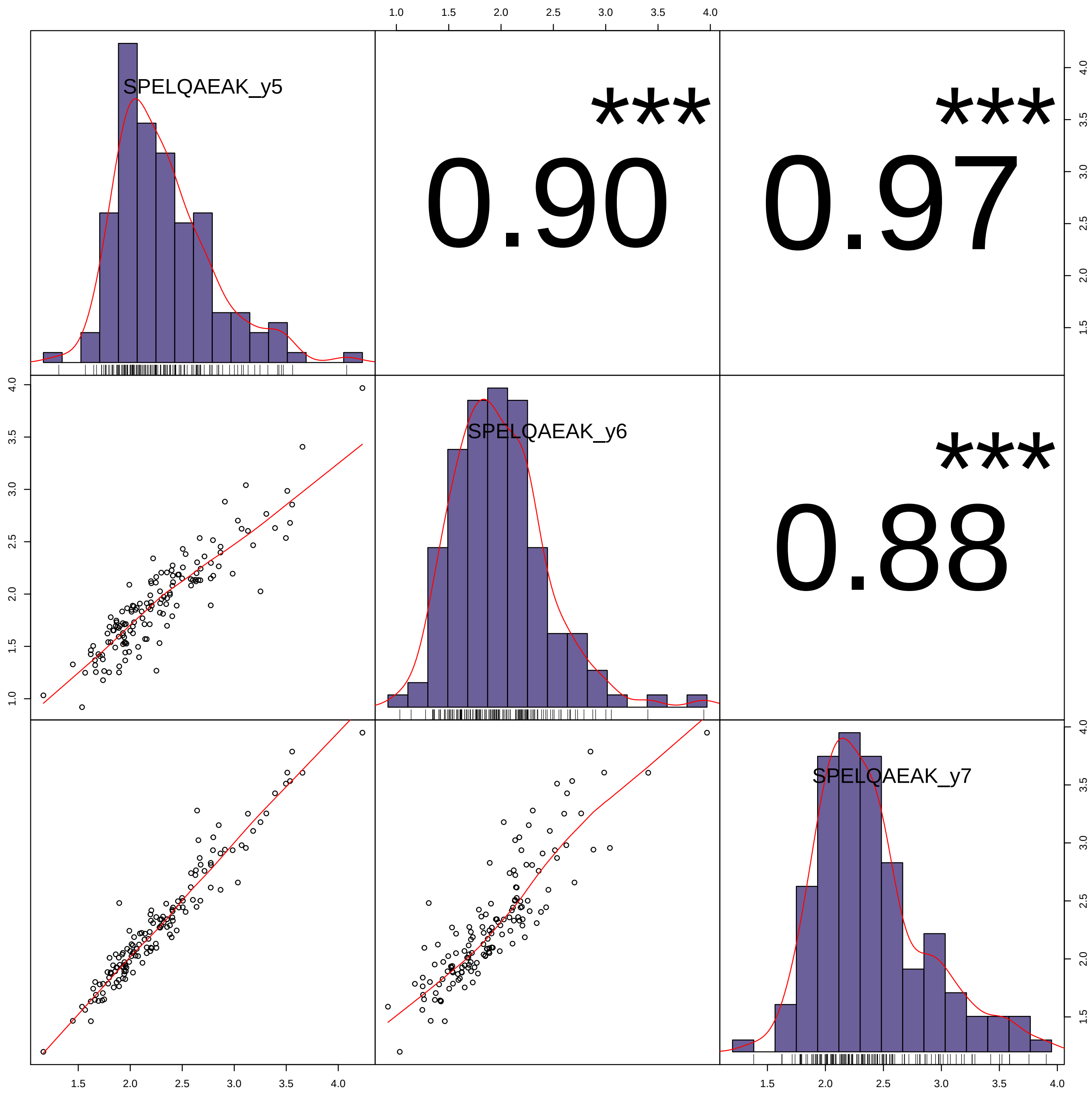

Supplementary Fig. S2c. Correlations between transitions for apoAII QPrEST HPRR4430020.

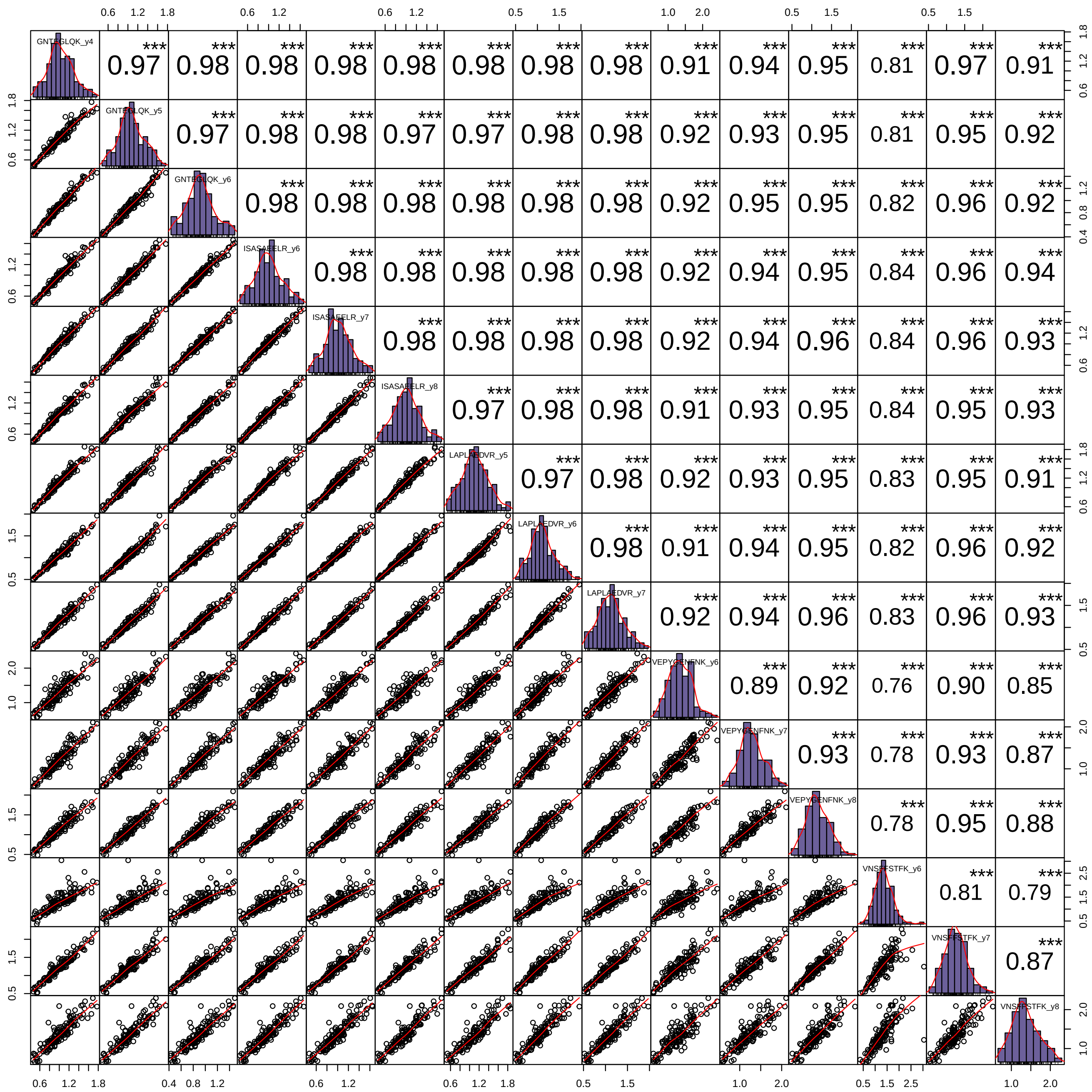

Supplementary Fig. S2d. Correlations between transitions for apoAIV QPrEST HPRR260124.

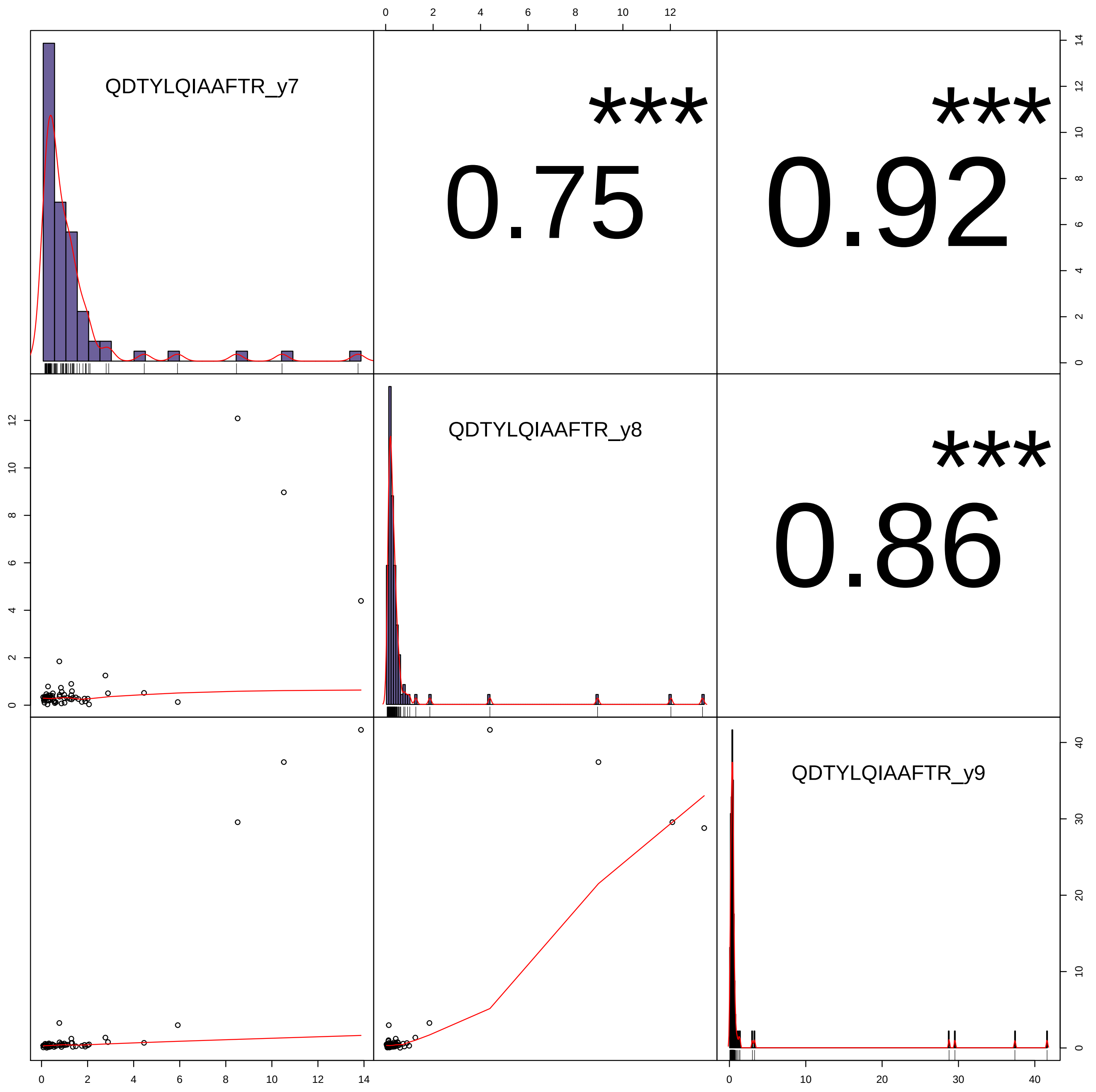

Supplementary Fig. S2e. Correlations between transitions for apoAV QPrEST HPRR3050130.

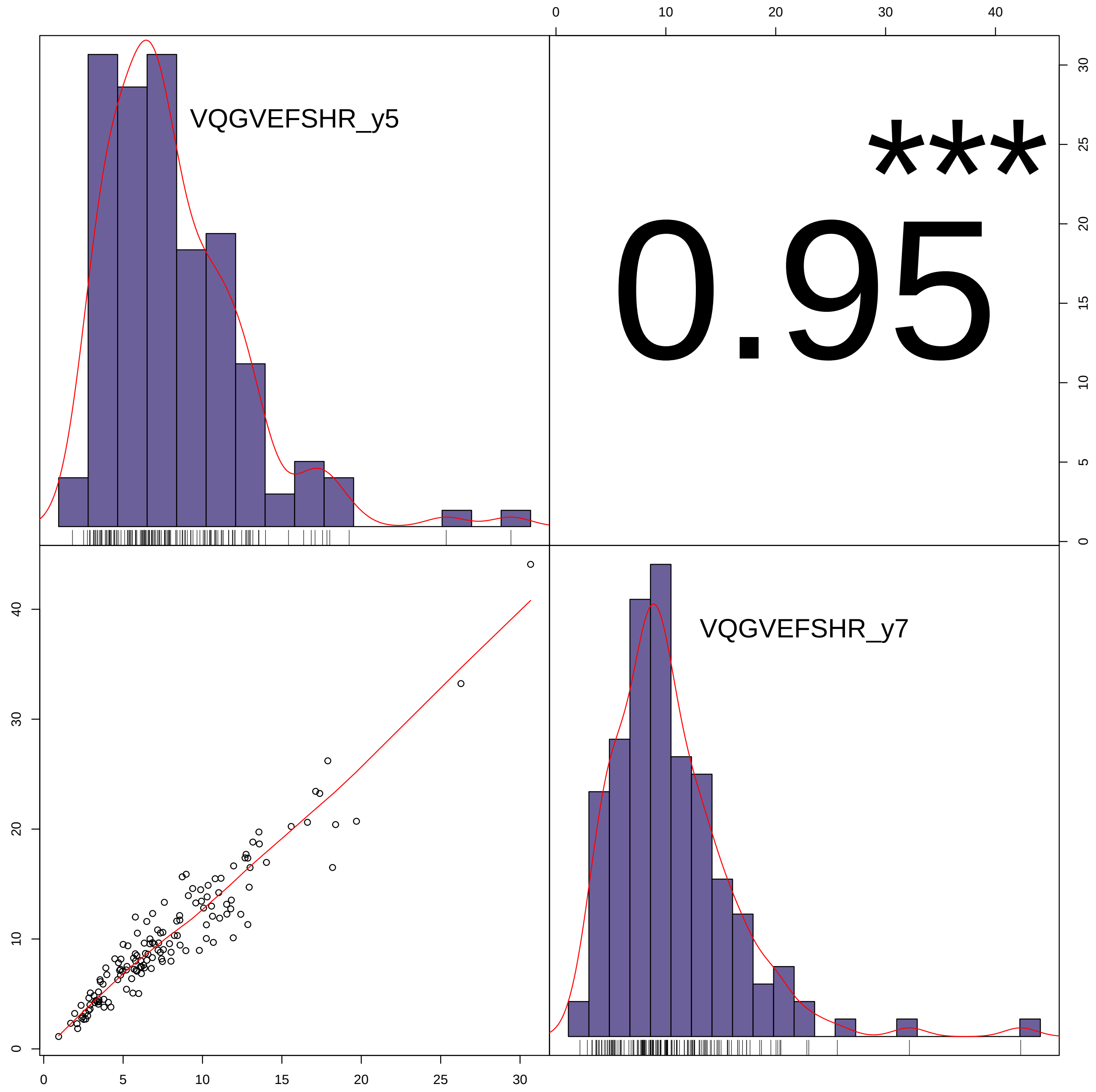

Supplementary Fig. S2f. Correlations between transitions for apoB QPrEST HPRR3720310.

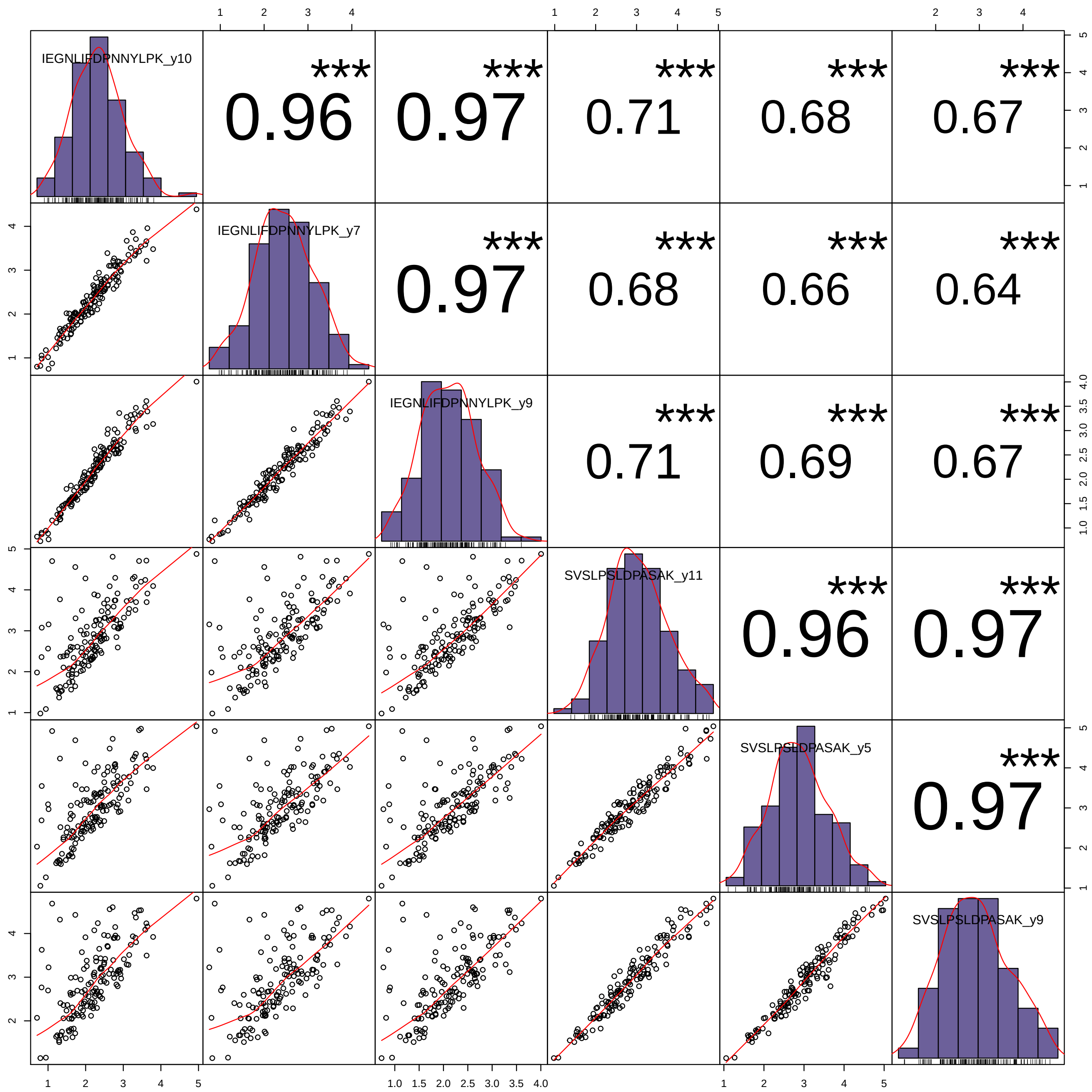

Supplementary Fig. S2g. Correlations between transitions for apoB QPrEST HPRR3720311.

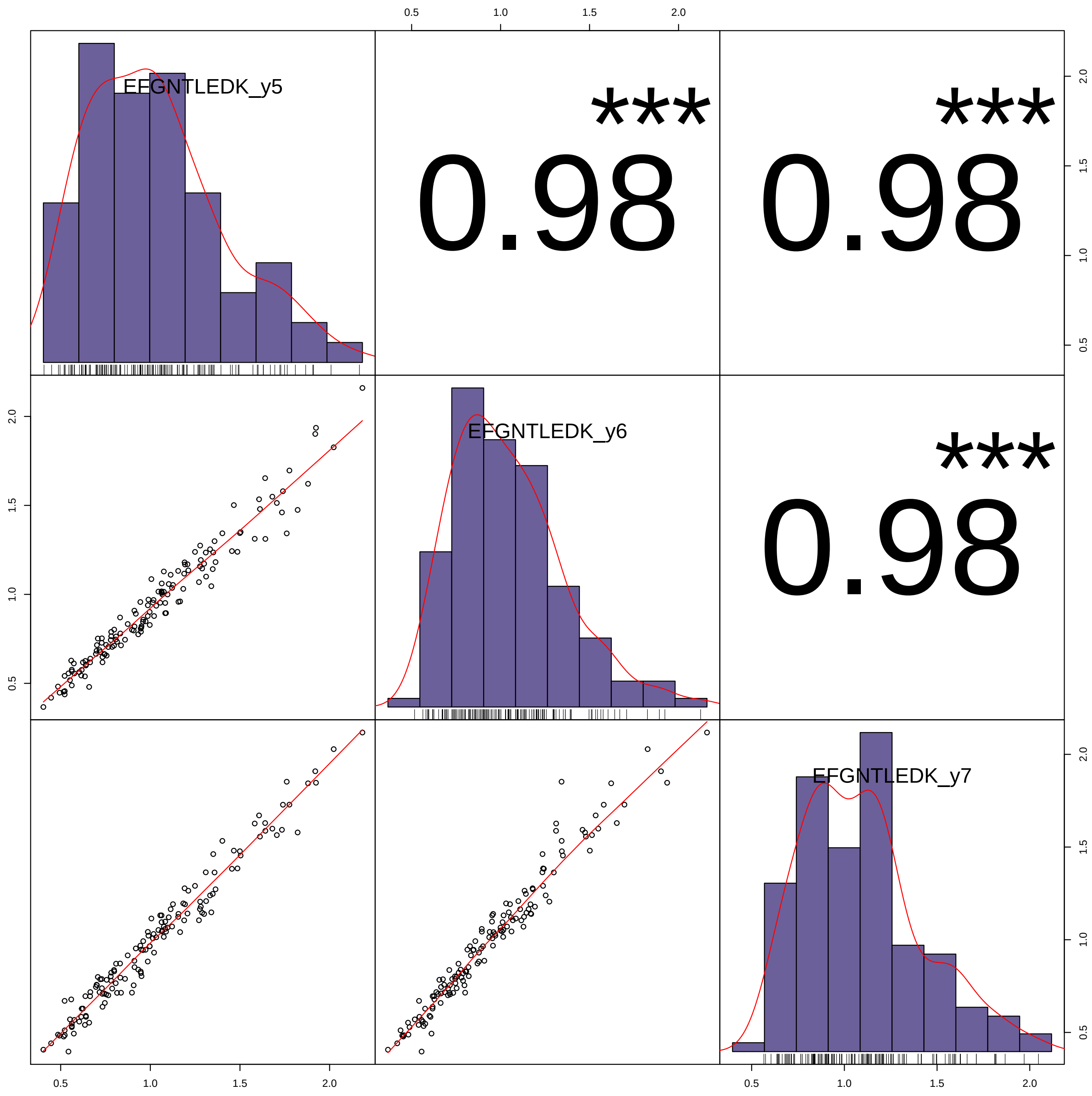

Supplementary Fig. S2h. Correlations between transitions for apoCI QPrEST HPRR3730489.

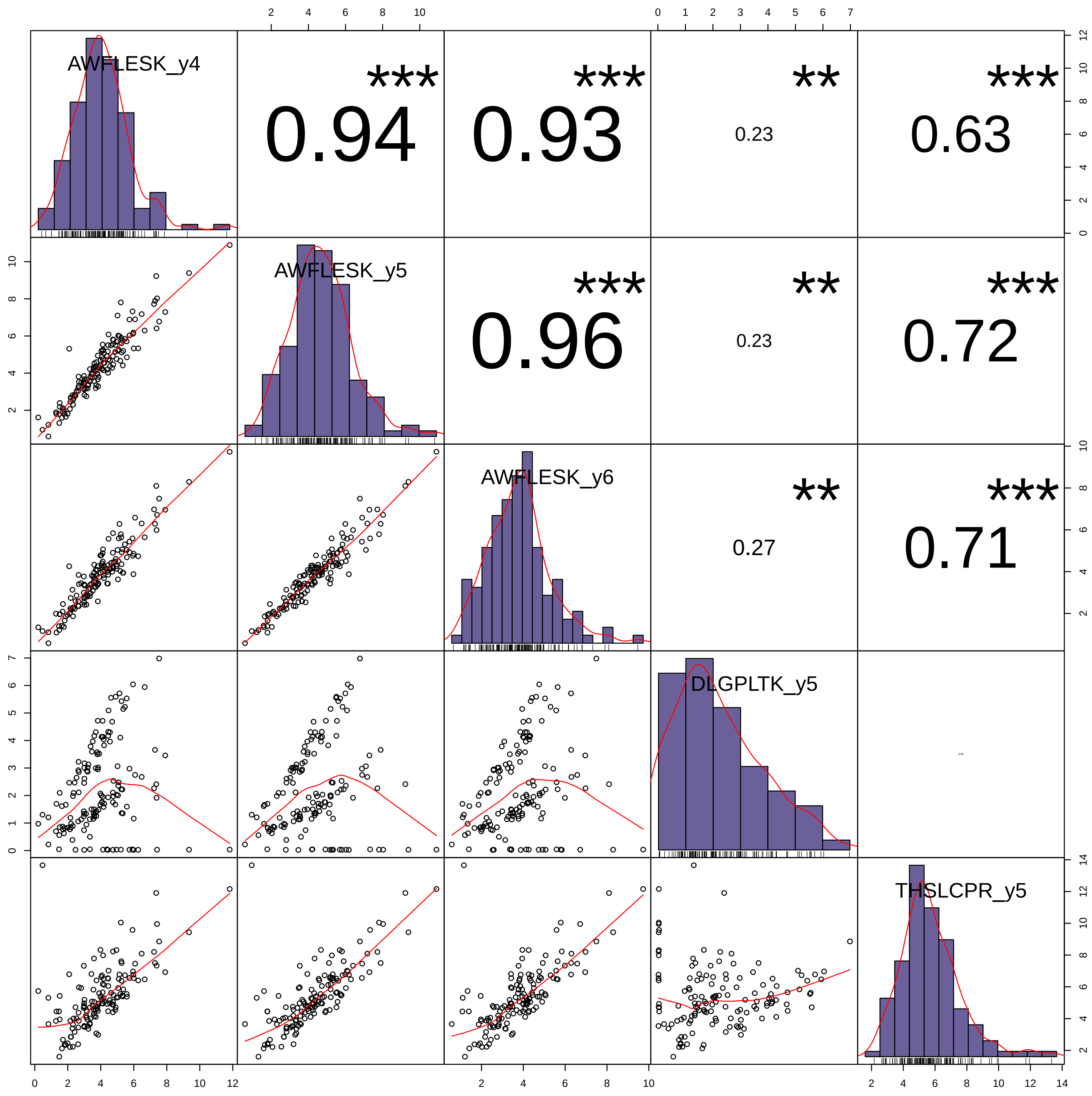

Supplementary Fig. S2i. Correlations between transitions for apoCIV QPrEST HPRR4130067.

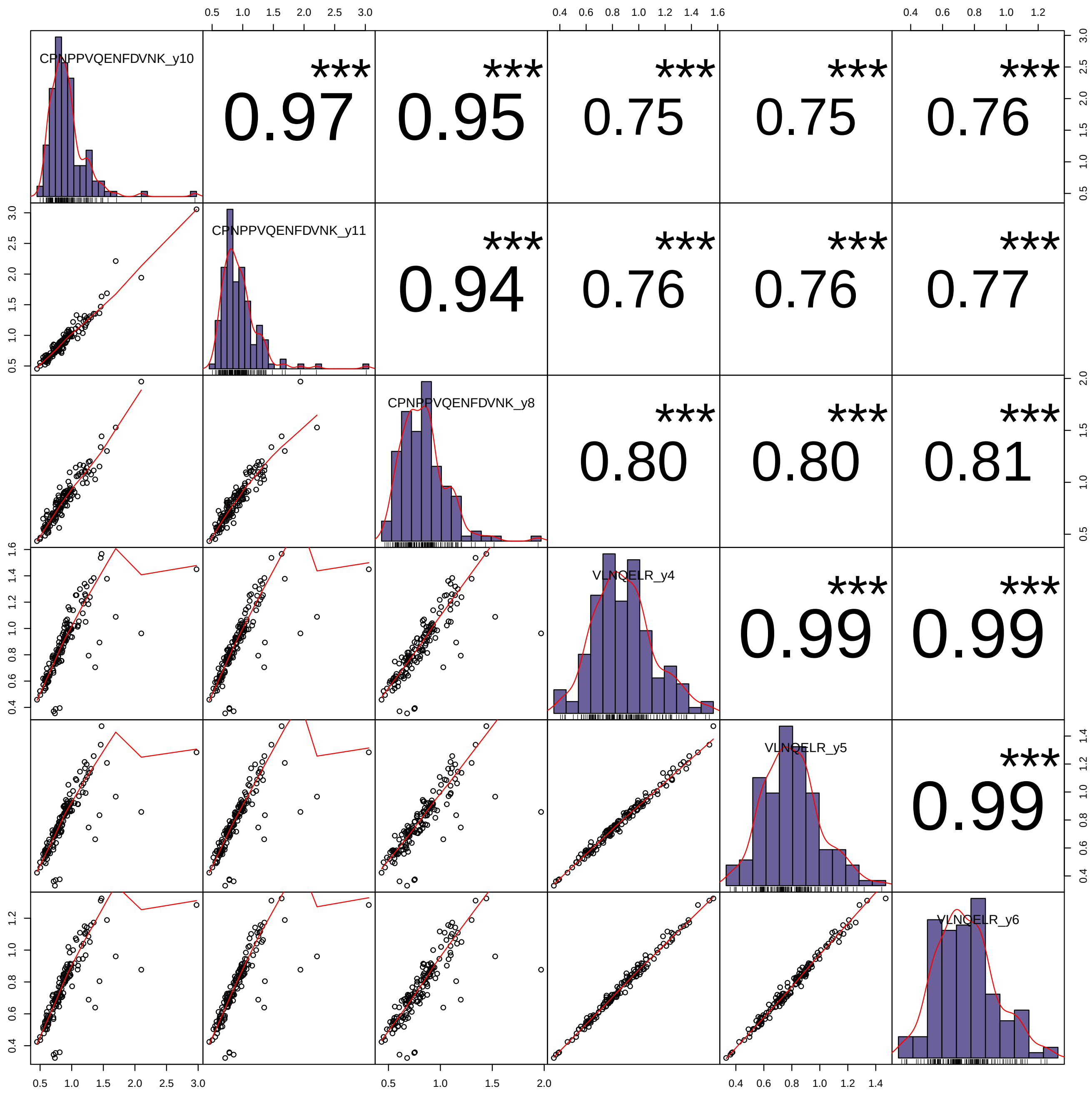

Supplementary Fig. S2j. Correlations between transitions for apoD QPrEST HPRR2760373.

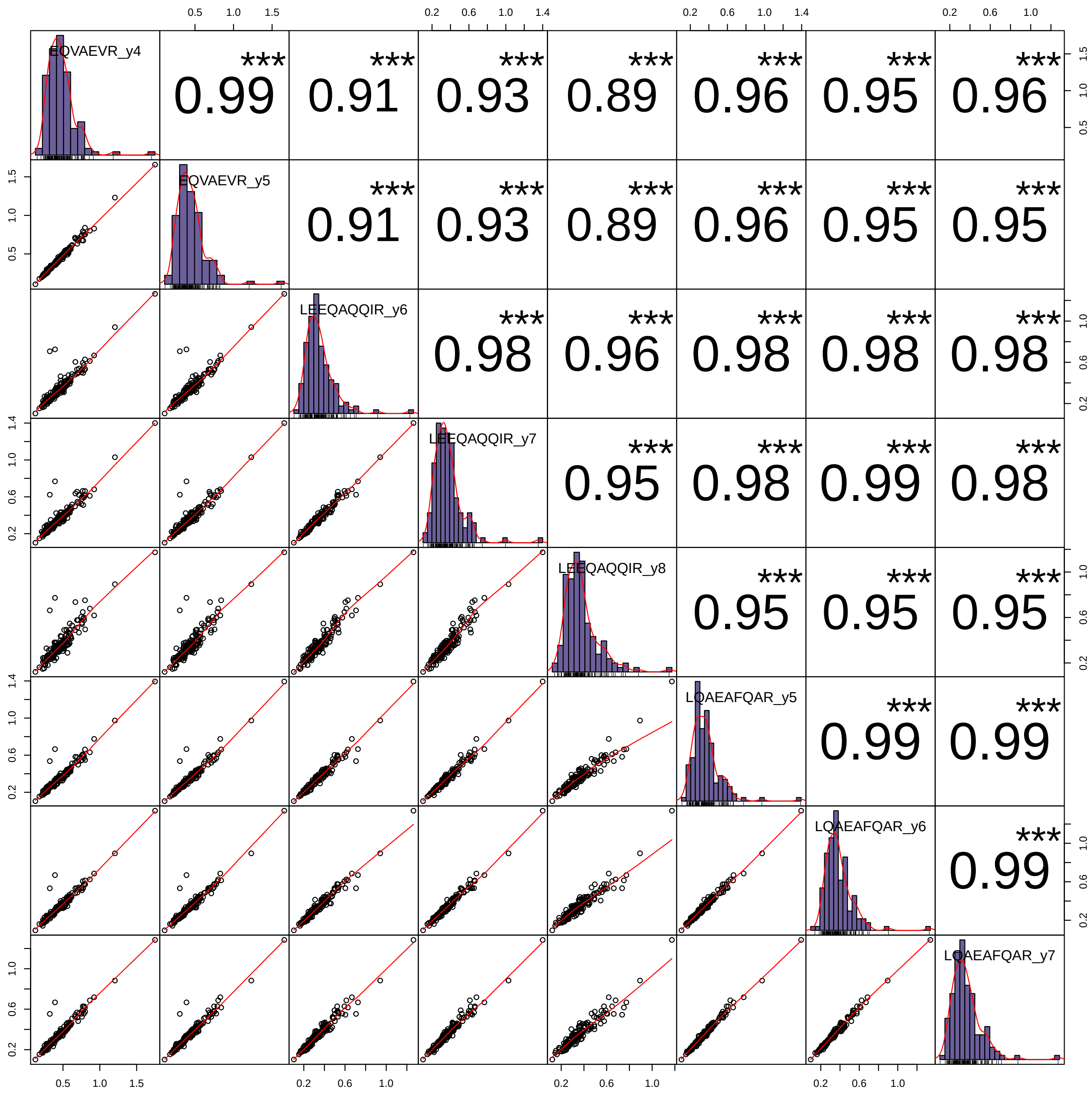

Supplementary Fig. S2k. Correlations between transitions for apoE QPrEST HPRR4200068.

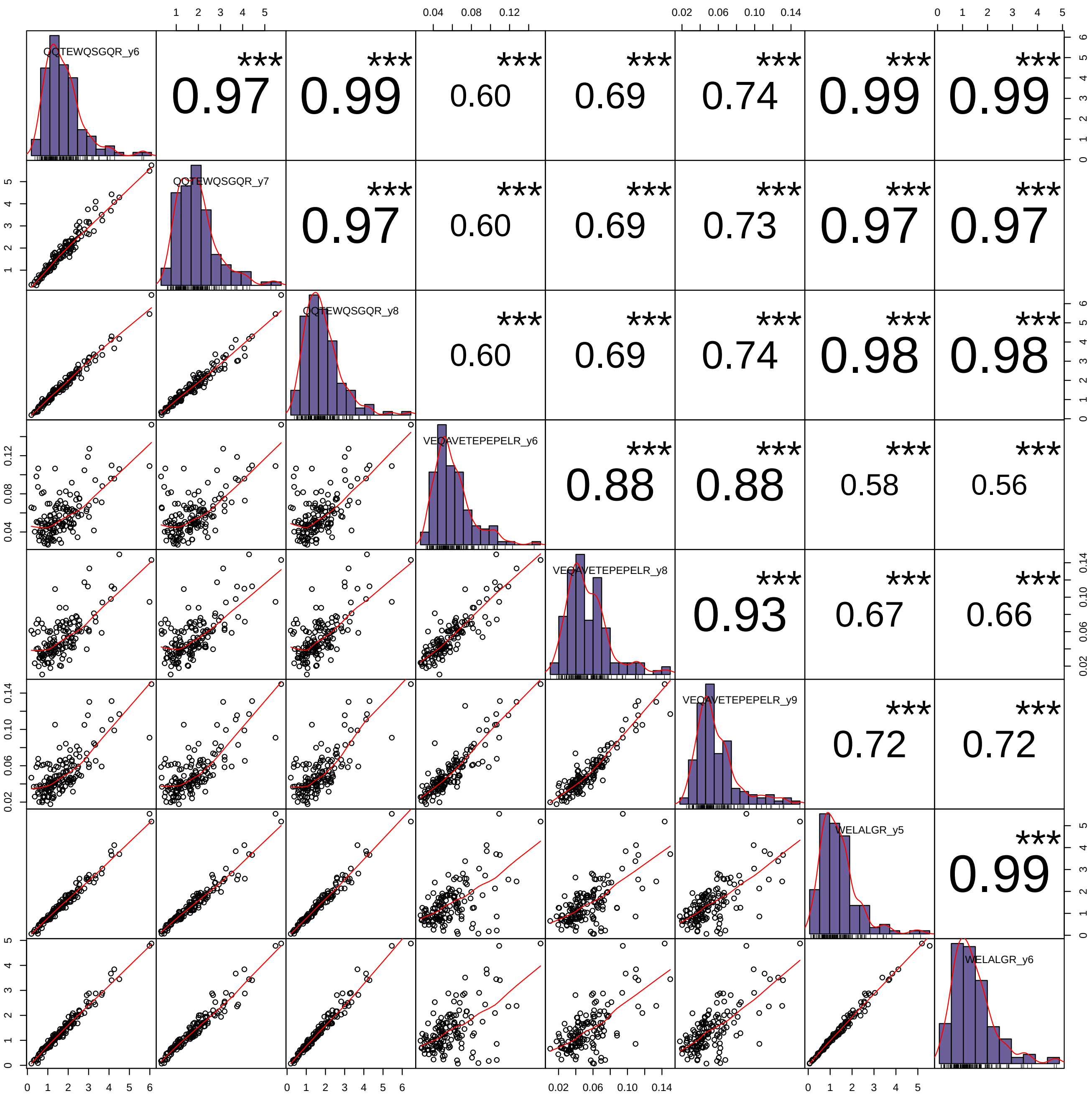

Supplementary Fig. S2I. Correlations between transitions for apoE QPrEST HPRR4340126.

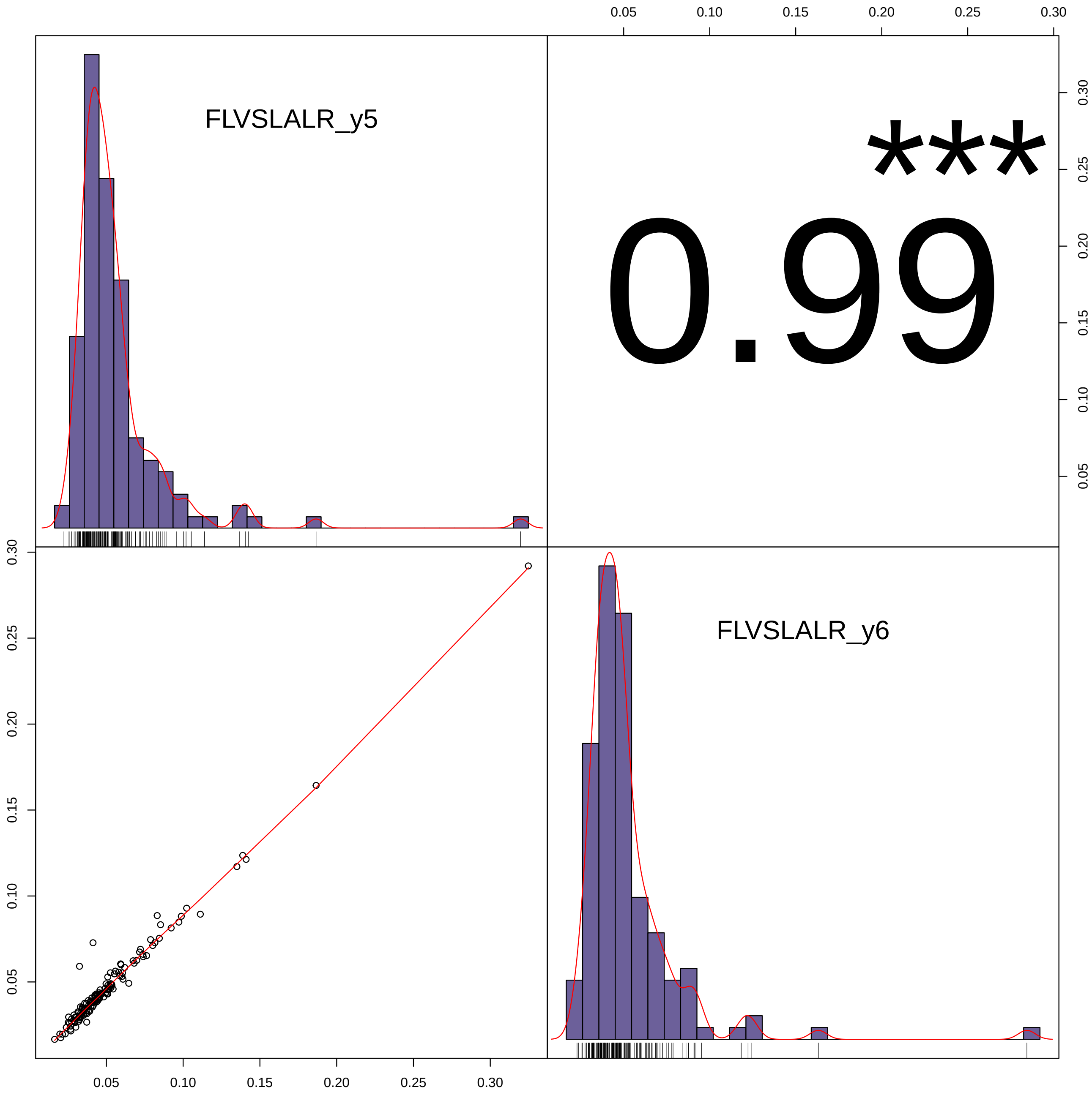

Supplementary Fig. S2m. Correlations between transitions for apoF QPrEST HPRR350023.

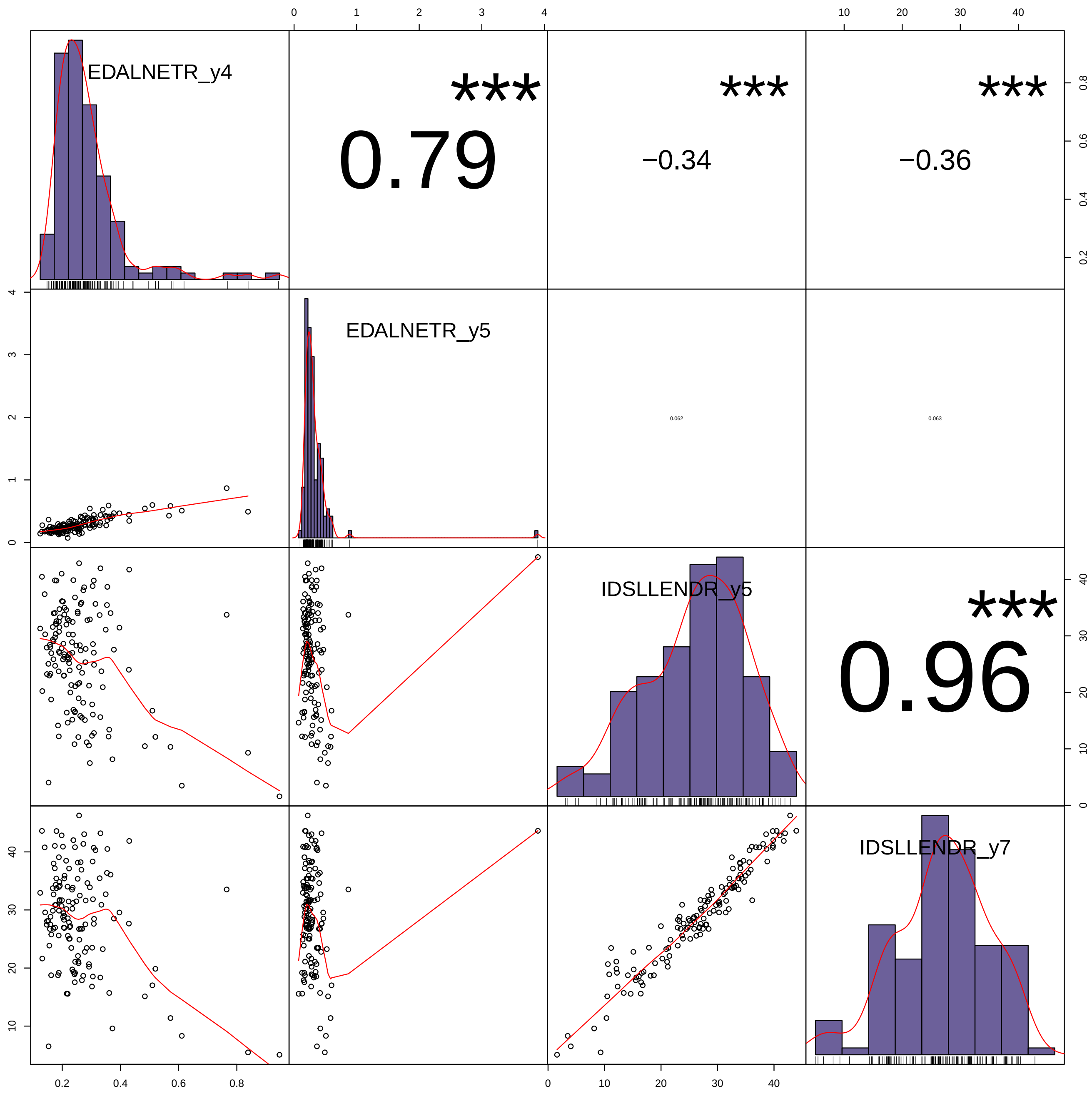

Supplementary Fig. S2n. Correlations between transitions for apoJ QPrEST HPRR4320626.

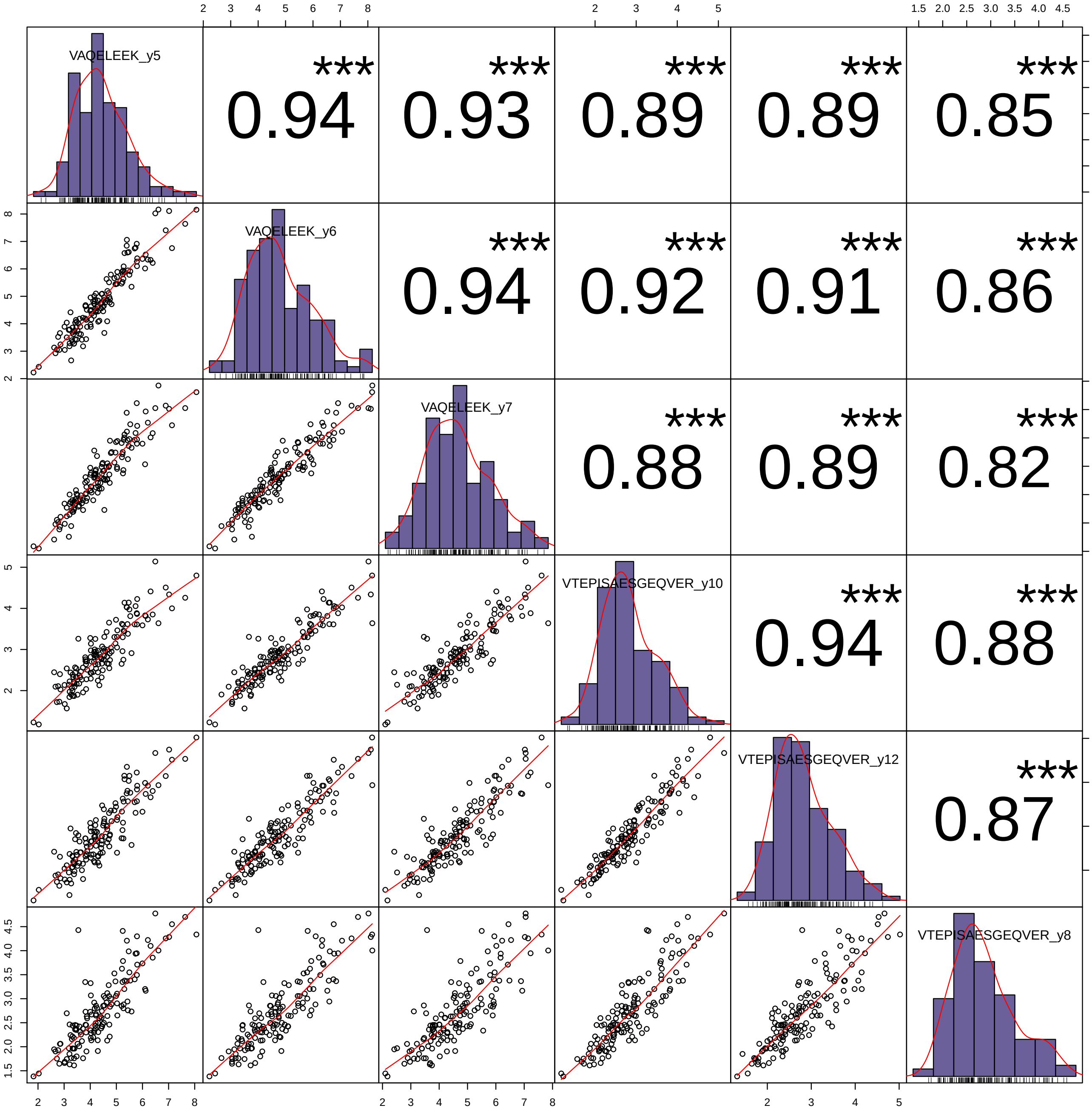

Supplementary Fig. S2o. Correlations between transitions for apoLI QPrEST HPRR350088.

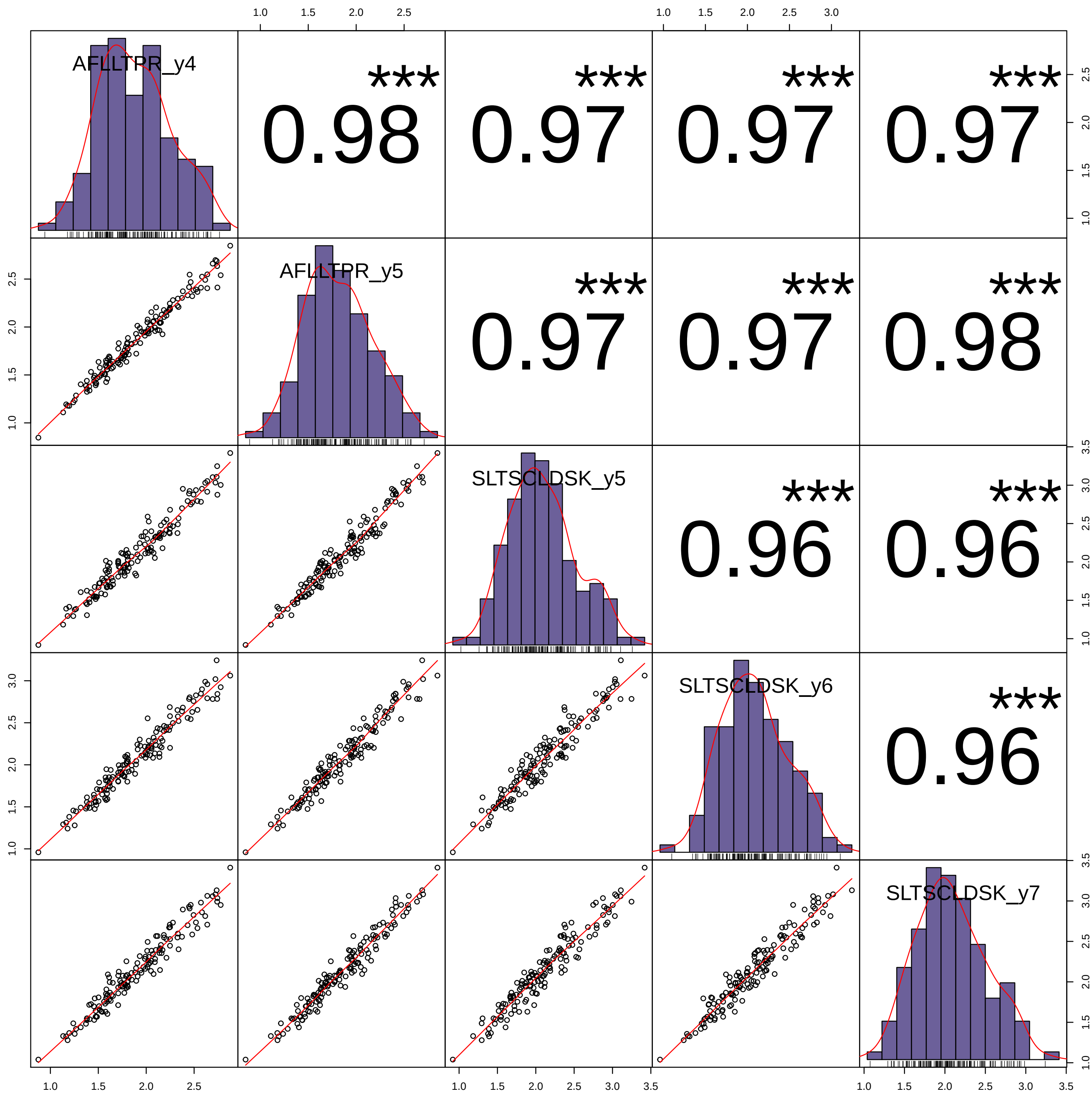

Supplementary Fig. S2p. Correlations between transitions for apoM QPrEST HPRR3340379.
